# Time-resolved proximity biotinylation implicates a porin protein in export of transmembrane malaria parasite effectors

**DOI:** 10.1242/jcs.260506

**Published:** 2023-10-18

**Authors:** David Anaguano, Watcharatip Dedkhad, Carrie F. Brooks, David W. Cobb, Vasant Muralidharan

**Affiliations:** ^1^ Department of Cellular Biology, University of Georgia, Athens, GA, USA; ^2^ Center for Tropical and Emerging Global Diseases, University of Georgia, Athens, GA 30602, USA

**Keywords:** Host-parasite interactions, Malaria, *Plasmodium falciparum*, Protein export, Translocons

## Abstract

The malaria-causing parasite, *Plasmodium falciparum* completely remodels its host red blood cell (RBC) through the export of several hundred parasite proteins, including transmembrane proteins, across multiple membranes to the RBC. However, the process by which these exported membrane proteins are extracted from the parasite plasma membrane for export remains unknown. To address this question, we fused the exported membrane protein, skeleton binding protein 1 (SBP1), with TurboID, a rapid, efficient and promiscuous biotin ligase (SBP1^TbID^). Using time-resolved proximity biotinylation and label-free quantitative proteomics, we identified two groups of SBP1^TbID^ interactors – early interactors (pre-export) and late interactors (post-export). Notably, two promising membrane-associated proteins were identified as pre-export interactors, one of which possesses a predicted translocon domain, that could facilitate the export of membrane proteins. Further investigation using conditional mutants of these candidate proteins showed that these proteins were essential for asexual growth and localize to the host–parasite interface during early stages of the intraerythrocytic cycle. These data suggest that they might play a role in ushering membrane proteins from the parasite plasma membrane for export to the host RBC.

## Teaser

**Summary:** A rapid time-resolved proximity biotinylation approach to identify novel candidates that facilitate the export of membrane proteins in the *P. falciparum*-infected red blood cell.

## INTRODUCTION

Malaria is a major global health issue, with an estimated 241 million cases and 627,000 deaths reported during 2021 (World Health Organization, 2021; https://www.who.int/teams/global-malaria-programme/reports/world-malaria-report-2021). This life-threatening disease is caused by *Plasmodium* parasites, belonging to the apicomplexan phylum. Among these parasites, *Plasmodium falciparum* is the most virulent and lethal, accounting for 95% of all malaria-related deaths (Moxon et al., 2020). Malaria symptoms include headaches, myalgia, high fevers, severe anemia, pulmonary and renal failure, vascular obstruction and cerebral damage. These disorders can persist even after parasite clearance and result from the proliferation of parasites within human red blood cells (RBCs) (Ashley et al., 2018; Moxon et al., 2020).

To establish infection during their intraerythrocytic cycle, *P. falciparum* parasites extensively remodel the morphology and physiology of the RBCs. This transformation requires the export of several hundred proteins (∼10% of the parasite proteome) across the unique parasitophorous vacuole (PV), a membrane surrounding the parasite, into the cytoplasm and membrane of RBCs (de Koning-Ward et al., 2016; Matthews et al., 2019a; Spielmann and Gilberger, 2015; Spillman et al., 2015). This process leads to increased permeability, loss of cell deformability and the formation of virulence-associated knobs at the RBC membrane (Desai, 2014; Maier et al., 2009). These multi-step transformations are crucial for parasite survival and pathogenesis, conferring *P. falciparum* its ability to maintain chronic infections in humans. A large fraction of exported proteins is recognizable by the presence of a 5-amino-acid motif, known as the *Plasmodium* export element or PEXEL (Hiller et al., 2004; Marti et al., 2004), whereas others lack a discernable primary sequence motif and are termed as PEXEL-negative exported proteins or PNEPs (Heiber et al., 2013). Most PNEPs possess a transmembrane (TM) domain that serves to target them to the endoplasmic reticulum (ER) and the secretory pathway (Heiber et al., 2013). Several of these PNEPs play critical roles in malaria pathogenesis, such as skeleton-binding protein 1 (SBP1) (Blisnick et al., 2000; Maier et al., 2007; Saridaki et al., 2009), membrane-associated histidine-rich protein (MAHRP1) (Spycher et al., 2003, 2008) and erythrocyte membrane protein 1 (PfEMP1) (Baruch et al., 1995, 1997; Su et al., 1995).

**Fig. 1.**
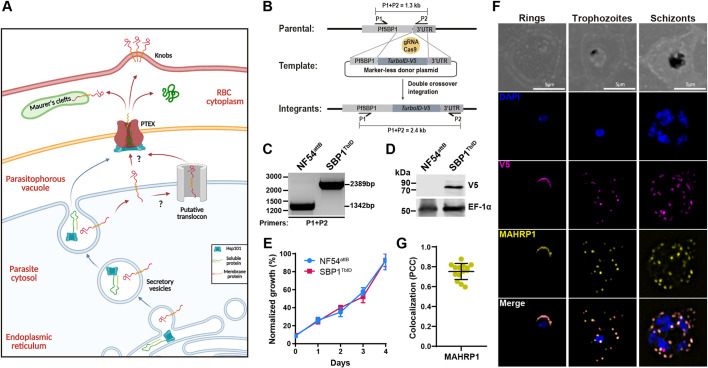
**Generation of SBP1^TbID^ mutants.** (A) Schematic of protein export. Membrane and soluble proteins are transported from the ER into the PV by secretory vesicles. Soluble proteins are released into the PV lumen after fusion of the secretory vesicle to the PPM. Membrane proteins, on the other hand, are inserted into the PPM and need to be extracted from the membrane by a putative translocon (PTEM) for further transport through the PV membrane to the RBC cytoplasm by the PTEX complex. Soluble and membrane proteins are transported to their final location in the infected RBC. Created by BioRender.com. (B) Schematic showing the integration of the repair plasmid used to tag the genomic loci of SBP1 with TurboID-V5. Cas9 introduces a double-stranded break at the C-terminus of the SBP1 locus. The repair plasmid provides homology regions for double-crossover homologous recombination, introducing TurboID and the V5 tag sequences. (C) PCR test confirming integration at the SBP1 locus. Amplicons were amplified from genomic DNA isolated from mutant and wild-type parasites. Primers were designed to amplify the region between the C-terminus and the 3′UTR of SBP1. (D) Western blot of parasite lysates isolated from the parental line (NF54^attB^) (Nkrumah et al., 2006) and a clone of SBP1^TbID^ (D10) probed with antibodies against V5 and EF1α (loading control). The protein marker sizes are shown on the left. (E) Growth of asynchronous SBP1^TbID^ parasites, compared to the parental line NF54^attB^, over 4 days via flow cytometry. 100% represents the highest value of calculated parasitemia. Representative of three biological replicates shown for each growth curve. Each data point represents the mean of three technical replicates; error bars represent the s.d. (F) IFA showing that SBP1^TbID^ localizes to the parasite periphery in the early-ring stage (left) and is exported to the MC in trophozoite (center) and schizont (right) stages. Asynchronous SBP1^TbID^ parasites were fixed with acetone and stained with specific antibodies. Images from left to right are phase-contrast, DAPI (nucleus, blue), anti-V5 (magenta), anti-MAHRP (yellow) and fluorescence merge. *Z-*stack images were deconvolved and projected as a combined single image. (G) Quantification of the colocalization of SBP1^TbID^ with MAHRP as determined by the Pearson's correlation coefficient. Three biological replicates were represented with four late-stage-parasite images from each replicate. Error bars represent s.d.

Exported membrane proteins are inserted into ER membrane during their synthesis (Gruring et al., 2012; Heiber et al., 2013; Spielmann et al., 2006). These membrane proteins are transported via vesicles from the ER and inserted into the parasite plasma membrane (PPM) when the transport vesicles fuse to the PPM (Gruring et al., 2012). Although it has been established that all exported proteins require the *Plasmodium* translocon of exported proteins (PTEX) complex to cross the PV membrane (PVM) (Beck et al., 2014; Elsworth et al., 2014), the mechanism by which membrane proteins are extracted from the PPM and delivered to the PTEX complex remains unknown. It has been postulated that a putative *Plasmodium* translocon of exported membrane proteins (which we term as PTEM) (Beck and Ho, 2021; Garten and Beck, 2021) is necessary for the extraction of membrane proteins from the PPM, either alone or in cooperation with the PTEX unfoldase HSP101 (Gabriela et al., 2022; Matthews et al., 2019b) (Fig. 1A). However, the identity of proteins in this putative PTEM complex is unknown, and no candidates have been identified using bioinformatic approaches within the *P. falciparum* genome. To address this knowledge gap, we attempted to utilize an unbiased proteomic approach to identify proteins potentially constituting the putative PTEM complex.

Immunoprecipitation (IP)-based proteomic approaches have been used previously to identify the exported-protein interacting complex (EPIC) at the PV, which is thought to be required for protein export (Batinovic et al., 2017). Similar approaches using *Plasmodium* exported proteins have identified stable complexes at the Maurer's clefts (MC), a parasite-generated protein sorting organelle in the RBC (Carmo et al., 2022; Jonsdottir et al., 2021; McHugh et al., 2020; Takano et al., 2019). However, the identification of the putative PTEM has proven elusive because its interaction with exported membrane proteins will be transient and therefore, unlikely to be captured using IP assays, which are heavily biased towards identifying stable complexes. Therefore, we used a rapid, proximity-labeling approach to attempt to identify a putative PTEM complex and to our knowledge, this approach has not yet been used in a time-resolved manner to capture transient interactions in the secretory pathway.

We chose to tag the endogenous SBP1 gene (PF3D7_0501300) with a new iteration of the promiscuous biotin ligase BirA, known as TurboID (generating SBP1^TbID^) (Branon et al., 2018). SBP1 is a PNEP with a single TM domain and is exported in early ring-stage parasites to the MC (Blisnick et al., 2000). TurboID is a highly efficient enzyme that is able to biotinylate proteins in close proximity within 10 min (Branon et al., 2018). Therefore, we hypothesized that SBP1^TbID^ will biotinylate proteins, even those transiently interacting with SBP1^TbID^ along the secretory pathway during its export to the MC. Given that SBP1^TbID^ should rapidly biotinylate proximal proteins, we further reasoned that we could differentiate early (pre-export) interactors from late (post-export) interactors of SBP1^TbID^. Our data show that the SBP1^TbID^ fusion protein is exported to the MC efficiently and with similar kinetics to another MC protein, MAHRP1. Crucially, SBP1^TbID^ is able to rapidly biotinylate proximal proteins prior to its export from the PV as well as after export at the MC. Using label-free quantitative proteomics, we compared pre-export interactors and post-export interactors of SBP1^TbID^. This approach led to the identification of two membrane-associated proteins that might play a role in the export of transmembrane malaria parasite effectors to the host RBC.

## RESULTS

### SBP1 fused to TurboID is exported to Maurer's clefts

Using CRISPR/Cas9 gene editing, we generated mutants of SBP1 (Fig. 1B), where the endogenous gene was tagged with the TurboID biotin ligase (SBP1^TbID^) (Branon et al., 2018; May et al., 2020). We chose TurboID because it is an optimized version of the biotin ligase BirA (Branon et al., 2018). TurboID is a highly active mutant of BirA with an increased biotinylation radius and faster biotinylation kinetics (Branon et al., 2018; May et al., 2020). PCR analysis of genomic DNA isolated from the SBP1^TbID^ parasite line showed the correct integration of the TurboID biotin ligase and a V5 tag at the endogenous locus of SBP1 (Fig. 1C). We detected expression of the SBP1^TbID^ in the mutant line at the expected size, but not in the parental line (Fig. 1D). To ensure that the expression of TurboID was not detrimental to the parasite, we observed the growth of SBP1^TbID^ and the parental parasite line (NF54^attB^) (Nkrumah et al., 2006), over several asexual cycles using flow cytometry (Fig. 1E). These data show no difference in the asexual growth of SBP1^TbID^ compared to that of the parental parasites, demonstrating that expression of TurboID or its fusion to SBP1 does not inhibit parasite growth.

SBP1 is an exported protein with a single TM domain synthesized in the parasite ER and transported to the MC in the RBC cytoplasm (Cooke et al., 2006; Mundwiler-Pachlatko and Beck, 2013). Therefore, we wanted to ensure that the fusion of TurboID to SBP1 did not inhibit its export to the MC. Using immunofluorescence microscopy (IFA), we tested whether SBP1^TbID^ colocalized with another MC resident protein, MAHRP1 (Spycher et al., 2008). These data show that SBP1^TbID^ was exported from the parasite to the MC and that it colocalized with MAHRP1 in trophozoite and schizont stage parasites (Fig. 1F,G). By contrast, in early ring stage parasites, these data show that SBP1^TbID^, as well as MAHRP1, localizes to the periphery of the parasite, probably in the PV prior to export (Fig. 1F). This has been previously observed by electron microscopy, where SBP1 accumulated in electron-dense regions within the PPM before being transported through the PV membrane (Iriko et al., 2020). These data suggest that SBP1, and possibly other MC-resident proteins, accumulate in the PV before being exported to the infected RBC. Together, the data show that tagging SBP1 with the TbID biotin ligase did not alter the asexual growth or development of the parasite, nor did it inhibit the export of SBP1 to the host RBC and MC (Fig. 1).

### Biotin-dependent proximity-labeling by SBP1^TbID^

**Fig. 2.**
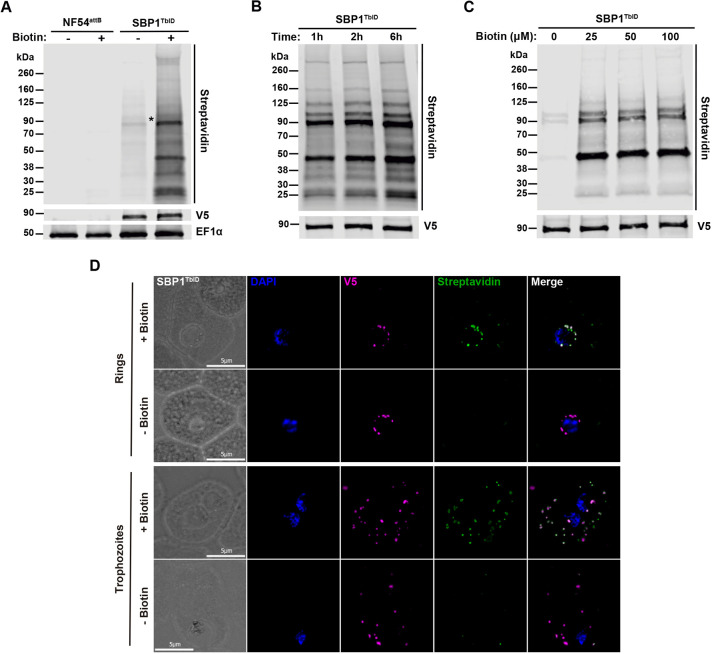
**Biotinylation of proximal proteins by TurboID_V5_-tagged SBP1.** (A) Western blot of parasite lysates isolated from the parental line NF54^attB^ and the mutant line SBP1^TbID^ incubated with or without biotin (50 μM) for 2 h. Samples were probed with antibodies against V5, EF1α (loading control) and fluorescent dye-labeled streptavidin. The protein marker sizes are shown on the left. (B) Western blot of parasite lysates isolated from the mutant line SBP1^TbID^ incubated with biotin (50 μM) for 1 h, 2 h and 6 h. Samples were probed with antibodies against V5 (loading control) and fluorescent dye-labeled streptavidin. The protein marker sizes are shown on the left. (C) Western blot of parasite lysates isolated from the mutant line SBP1^TbID^ incubated with different concentrations of biotin (0, 25, 50 and 100 μM) for 1 h. Samples were probed with antibodies against V5 (loading control) and fluorescent dye-labeled streptavidin. The protein marker sizes are shown on the left. (D) IFA showing SBP1^TbID^ biotinylated proteins during their export out of the parasite (top panels) and at their final location at the Maurer's clefts (bottom panels). Synchronous SBP1^TbID^ parasites were fixed with acetone after 2 h of incubation with biotin (50 μM) and stained with specific antibodies. Images from left to right are phase contrast, DAPI (nucleus, blue), anti-V5 (magenta), streptavidin (green) and fluorescence merge. Z-stack images were deconvolved and projected as a combined single image. All results are representative of three experimental repeats.

Given that our data show that the SBP1^TbID^ fusion protein was exported to MC, we wanted to examine the capacity of TurboID to biotinylate proximal proteins in SBP1^TbID^ parasites. TurboID is an extremely efficient enzyme and we found it could utilize the minimal amount of biotin present in the medium used to grow SBP1^TbID^ parasites (Fig. S1). The normal asexual development of *P. falciparum* does not require biotin (Dellibovi-Ragheb et al., 2018). To test whether SBP1^TbID^ biotinylation is dependent upon the presence of exogenous biotin, we analyzed protein extracts of asynchronous parasites in the presence or absence of biotin by streptavidin blotting. We observed that efficient biotinylation of proximal proteins occurred only in the presence of biotin in SBP1^TbID^ parasites (Fig. 2A). Self-biotinylation in SBP1^TbID^ parasites was observed in the presence or absence of biotin (Fig. 2A, lane 3 and 4, see asterisk), in agreement with what has been previously reported when tagging proteins with TurboID (Branon et al., 2018; Larochelle et al., 2019). No endogenous biotinylation was detected in the parental line NF54^attB^, showing that biotinylation occurs only when TurboID is being expressed by the parasite line (Fig. 2A, lanes 1 and 2). These data show that SBP1^TbID^ efficiently biotinylated proteins and its activity is dependent upon the presence of biotin in the growth medium.

TurboID is a highly active enzyme (Branon et al., 2018) that offers the possibility of rapid and time-resolved labeling approaches in contrast to previous proximity-labeling methods with much longer incubation times, usually greater than 12 h (Kim et al., 2016; Kudyba et al., 2019; Roux et al., 2012). Thus, we wanted to assess the biotinylation activity of SBP1^TbID^ and test whether this fusion protein can rapidly biotinylate proximal proteins. SBP1^TbID^ parasites were incubated with biotin for 1, 2 or 6 h, and the biotinylation of proteins was observed via western blots probed with streptavidin (Fig. 2B). We also tested biotinylation in response to different concentrations of biotin (25, 50 and 100 μM; Fig. 2C). Biotinylated proteins were observed at all time points and biotin concentrations, and the observable difference in the extent of protein biotinylation between the time points and concentrations was minimal (Fig. 2B,C).

The SBP1^TbID^ fusion protein has to traverse several membranes during its export to the MC, and therefore, it is likely to unfold and then refold during this transport process. In the case of exported membrane proteins, it is not known whether they are kept unfolded during their transport, although all proteins have to unfold while crossing the PV membrane using the PTEX complex at the PV membrane (Beck et al., 2014; Elsworth et al., 2014; Ho et al., 2018). Furthermore, to our knowledge, TurboID has not yet been utilized in a time-resolved manner to identify transient interactors as proteins are transported through the secretory pathway. Therefore, we wanted to determine whether SBP1^TbID^ parasites could biotinylate proteins proximal to SBP1 at different cellular locations during the export of SBP1^TbID^ from the parasite ER to the MC. Synchronized early ring and trophozoite stage parasites were observed by IFAs after the addition of biotin for 2 h. We observed biotinylation at the parasite periphery, possibly when SBP1^TbID^ accumulates at the PV (Iriko et al., 2020) (Fig. 2D, top panels). Biotinylation was also observed when SBP1^TbID^ had been exported to the MC (Fig. 2D, bottom panels). The observed biotinylation was dependent upon the addition of biotin. Together, these data demonstrate that SBP1^TbID^ is highly active, efficient, rapid and labels proximal proteins at different subcellular locations during its export from the parasite ER to the final location at the MC (Fig. 2).

### Early interactors of SBP1^TbID^ identified by proximity labeling

**Fig. 3.**
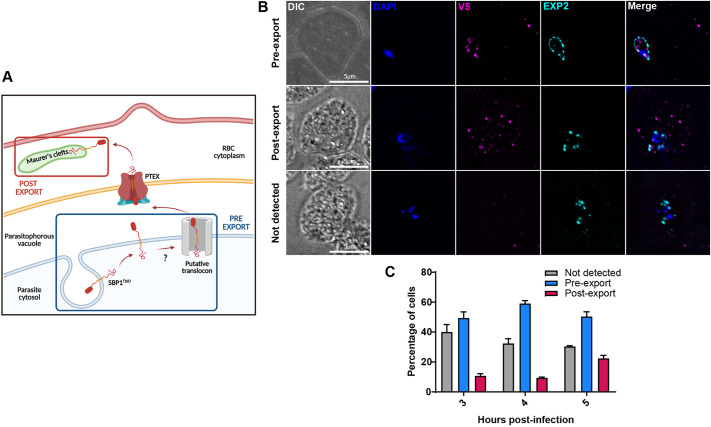
**Timing of SBP1 export.** (A) Schematic of the export of proteins in *P. falciparum* highlighting the locations where proteins biotinylated by SBP1^TbID^ will be harvested. Created with BioRender.com. (B) IFA showing the different localizations of SBP1^TbID^ during its export at early ring stages (3–5 hpi). Tightly synchronous SBP1^TbID^ parasites were fixed with acetone at 3 h, 4 h and 5 h post-infection, and stained with specific antibodies. Images from left to right are phase-contrast, DAPI (nucleus, blue), anti-V5 (magenta), EXP2 (PV marker, cyan), and fluorescence merge. *Z*-stack images were deconvolved and projected as a combined single image. (C) Quantification of the events observed in (B). Events were scored based on the localization of SBP1^TbID^ with respect to the PV marker EXP2. A total of 50 parasites were scored for each time point. *n*=3 biological replicates; error bars represent s.d.

Since our data show that SBP1^TbID^ biotinylated proximal proteins during its transit via the secretory pathway to the RBC cytoplasm (Fig. 2C), we next wanted to identify the *P. falciparum* effectors that interact with SBP1 at the host–parasite interface. To do so, we wanted to define the kinetics of SBP1^TbID^ transport from its site of synthesis in the parasite ER to its export to the MC and test whether we could reproducibly detect SBP1 at the host–parasite interface. As described above (Figs 1E, 2D), SBP1^TbID^ and proteins biotinylated by SBP1^TbID^ could be detected at the parasite–RBC interface. To assess whether we could reproducibly observe SBP1^TbID^ within the parasite prior to its export to the host RBC, we used tightly synchronized cultures and observed the subcellular localization of SBP1^TbID^ with respect to EXP2, a PVM-resident protein (Charnaud et al., 2018; Garten et al., 2018), at different time points after parasite invasion. SBP1 has been detected at the MCs as early as 4–6 h post-invasion (hpi) (Grüring et al., 2011); therefore, we observed the subcellular location of SBP1^TbID^ in parasites at 3, 4 and 5 hpi. In some SBP1^TbID^ parasites, SBP1 was either not detectable or not expressed (Fig. 3B, top panels). As expected, we found parasites where SBP1^TbID^ was within the PV periphery and others where the protein was already exported to the RBC cytoplasm (Fig. 3B, mid and bottom panels). We quantified these three events over several biological replicates. At 4 hpi, SBP1^TbID^ was not expressed in ∼30% of the parasites, exported in ∼10% of observed parasites, and at the host–parasite interface in the vast majority (60%) of all parasites (Fig. 3C). These data showed us that harvesting proteins biotinylated by SBP1^TbID^ at the host–parasite interface was feasible.

To identify early interactors of SBP1, especially those at the host–parasite interface, we opted for a quantitative and comparative approach. We wanted to differentiate these early interactors from SBP1 interactors at the MC, which have been previously identified (Takano et al., 2019), as well as those being co-transported with SBP1 to the MC. We hypothesized that using label-free quantitative proteomics and comparing interactors isolated from 4 hpi and 20 hpi would allow us to identify the early interactors of SBP1. By 20 hpi, all SBP1 is at the MCs and no more SBP1 is synthesized (Fig. 2D; McMillan et al., 2013). Label-free proteomics has been shown to offer a large dynamic range and high proteome coverage for the identification of biotinylated proteins (Larochelle et al., 2019; Lobingier et al., 2017; Mair et al., 2019; Santos-Barriopedro et al., 2021).

First, tightly synchronized late-stage schizonts were collected. These parasites were then split into two samples. One sample was incubated with biotin for 4 h until 4 hpi (Fig. 3A, blue box), after which it was collected for further processing. Based on our data, which indicate that SBP1^TbID^ is predominantly localized at the host–parasite interface during the 4-h ring stage (Fig. 3C), parasites were incubated with biotin for 4 h to maximize the labeling of proximal proteins and capture a larger fraction of the pre-export interactors. To collect the post-export sample, SBP1^TbID^ parasites were allowed to develop until 16 hpi, as by this time, all SBP1 is localized to the MC and is no longer synthesized. Thus, the other sample was allowed to develop without biotin for 16 h and then incubated with biotin for 4 h until 20 hpi (Fig. 3A, red box).

**Fig. 4.**
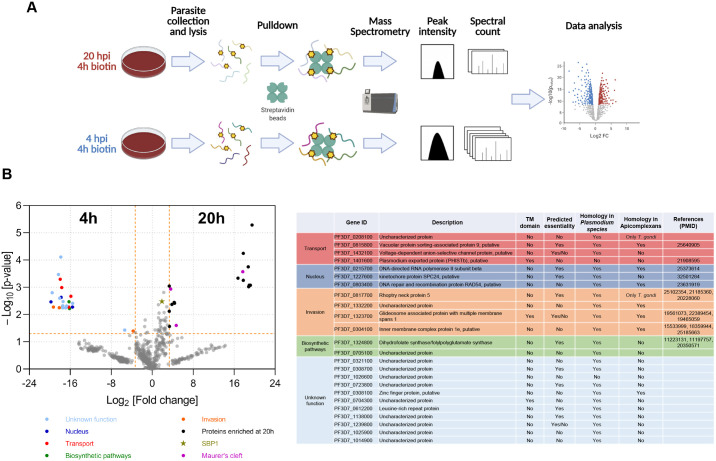
**Time-resolved proximity biotinylation to Identify the proteins interacting with SBP1 during its export.** (A) Schematic of the experimental design for time-resolved biotinylation (with 50 µM biotin) and proteomics to identify pre-export and post-export interactors of SBP1^TbID^. Created with BioRender.com. (B) Interactors enriched at 4 hpi (*P*-value plotted as function of fold change between the two time points). Proteins with *P*≤0.05 and more than 10-fold change are identified as SBP1^TbID^ interactors. *n*=3 biological replicates. (C) A summary table of the putative interactors of SBP1^TbID^ at 4 hpi grouped by their putative functions is shown. All proteins identified are in Table S1.

Biotinylated proteins were isolated from parasite lysates using streptavidin-affinity pulldown. The streptavidin-captured proteins were identified via mass spectrometry (MS) and quantified over several biological replicates (Larochelle et al., 2019; Lobingier et al., 2017; Mair et al., 2019) (Fig. 4A; Table S1). In total, 1122 proteins were identified in at least one of the replicates (Table S1). We then compared the proteins identified in the 4 h sample with those identified in the 20 h sample (Fig. 4B). We defined the putative pre-export interactors of SBP1 from our dataset using three stringent criteria. Proteins that exhibited more than 10-fold enrichment compared to the 20 h samples, with a *P*-value cut-off of 0.05 and were present in all three biological replicates, were considered as differentially labeled interactors at 4 hpi. Using these criteria, we identified 24 protein candidates as putative pre-export interactors of SBP1^TbID^ during its transport at the parasite–RBC interface (Fig. 4B). Among the identified proteins, only two were specific to *P. falciparum*, whereas the remaining 22 had homologs in other *Plasmodium* species. Interestingly, the majority of proteins with unknown functions were exclusive to *Plasmodium*, and nine proteins had homologs in other Apicomplexans (Fig. 4B; Table S2).

The identified proteins were classified into subgroups based on their predicted functions and subcellular locations (Amos et al., 2022). Of the 24 identified proteins, 11 were uncharacterized proteins with no predicted function. As expected, this approach identified proteins known to be involved in protein and vesicle transport (5/24). One of the statistically significant interactors of SBP1 was EXP3 (3-fold enriched at 4 hpi), which has been localized to the PV and functions in protein export (Batinovic et al., 2017). The experiment worked as designed because SBP1 (star, Fig. 4B) and other MC-localized, as well exported, proteins were also identified but were not enriched at either time point or enriched in the 20 hpi samples (Fig. 4B). Identification of exported proteins, including MC proteins, only in the post-export (20 hpi) samples further suggests that the proteomic approach using SBP1^TbID^ worked as designed. Together, these data showed that our approach successfully identified a group of proteins differentially biotinylated by SBP1^TbID^ prior to its export to the MC.

### Early interactors of SBP1^TbID^ localize to the host-parasite interface

Given that we were interested in identifying proteins that facilitate the export of transmembrane proteins through the PPM, we reasoned that membrane-associated proteins among pre-export SBP1 interactors could function in this role. Thus, based on membrane association, high statistical score, and fold enrichment, we selected the Glideosome-associated protein with multiple membrane spans 1 (GAPM1; PF3D7_1323700) as one putative candidate. GAPM1 is a membrane protein associated with the biogenesis of the inner membrane complex (IMC) in asexual and sexual stages. GAPM1, as part of the IMC, is suggested to have a role in merozoite invasion (Bullen et al., 2009; Kono et al., 2012, 2013). Using these criteria, another putative candidate was the channel protein Voltage-dependent anion-selective channel protein (VAC; PF3D7_1432100). VAC is a soluble protein with a translocon of the outer mitochondrial membrane (TOM40) domain but no mitochondria-targeting signal. Recent work has shown that VAC is a putative membrane protein that does not localize to the mitochondrial membrane (Lamb et al., 2022). Nothing is known about the function of VAC in *P. falciparum*.

To characterize these proteins, we used CRISPR/Cas9 gene editing to generate the conditional mutants, termed VAC^apt^ and GAPM1^mNG-apt^. In these parasite lines, their endogenous loci were tagged with the *tetR* aptamer system, which results in anhydrotetracycline (aTc)-dependent expression of the protein (Fig. S2A,B; Rajaram et al., 2020). PCR analysis of genomic DNA from VAC^apt^ and GAPM1^mNG-apt^ parasite lines showed correct integration of the knockdown system at the endogenous loci (Fig. S2C). To assess the efficiency of the knockdown system, we measured protein expression in the presence or absence of aTc by western blotting. For both proteins, there is a clear reduction of protein expression (Fig. S2D), which in the case of GAPM1^mNG-apt^ was detrimental, as parasites were not able to progress into a second life cycle. Knockdown of VAC inhibited the asexual expansion of VAC^apt^ parasites (Fig. S2E).

**Fig. 5.**
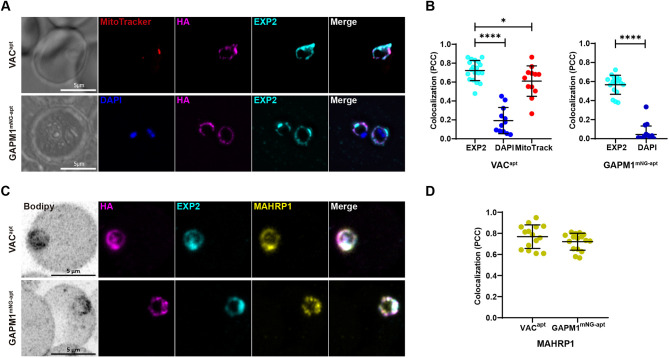
**VAC^apt^ and GAPM1^mNG-apt^ localize to the host–parasite interface.** (A) Representative IFAs showing VAC^apt^ and GAPM1^mNG-apt^ localization in early ring stages (4 hpi). Tightly synchronous parasites were fixed with PFA (VAC^apt^) and acetone (GAPM1^mNG-apt^) and stained with specific antibodies. Images of VAC^apt^ from left to right are phase-contrast, anti-HA (magenta), anti-EXP2 (PV, cyan), MitoTracker (mitochondria, red) and fluorescence merge. Images of GAPM1^mNG-apt^ from left to right are phase-contrast, DAPI (nucleus, blue), anti-HA (magenta), anti-EXP2 (PV, cyan) and fluorescence merge. *Z*-stack images were deconvolved and projected as a combined single image. (B) Quantifying the colocalization of VAC and GAPM1^mNG^ with respect to EXP2, MitoTracker (Mitochondrial marker) and DAPI, and to EXP2 and DAPI, respectively, determined by the Pearson's correlation coefficient. Three biological replicates are represented with six parasite images from each replicate for EXP2, and three parasite images for MitoTracker and DAPI. Error bars represent s.d. ****P<0.05 (unpaired, two-tailed Student's *t*-test). (C) Representative IFAs imaged by Airyscan microscopy showing VAC^apt^ and GAPM1^mNG-apt^ localization in early-ring stages (4 hpi). Tightly synchronous parasites were fixed with PFA and stained with BODIPY TRc (grayscale), anti-HA (magenta), anti-EXP2 (cyan), and anti-MAHRP1 (yellow). Z stack images were projected as a combined single image. (D) Quantifying the colocalization of VAC and GAPM1^mNG^ with respect to MAHRP1 as determined by the Pearson's correlation coefficient. Three biological replicates were represented with five parasite images from each replicate. Error bars represent standard deviation. ns, not significant (unpaired, two-tailed Student's *t*-test).

Our data confirms that GAPM1^mNG^ localizes to the IMC in schizonts (Fig. S3) (Bullen et al., 2009). However, the localization the GAPM1 post-invasion in the early ring stages is not known. Similarly, the subcellular localization of VAC during the early stages of the asexual life cycle was unknown. The proteomic data suggest that these proteins are in close proximity to SBP1^TbID^ when SBP1 is in the PV (Fig. 4B). Therefore, we used IFAs to localize both proteins in tightly synchronized parasites at 4 hpi with respect to the PV marker EXP2. VAC^apt^ localizes to the parasite periphery and is closely juxtaposed with the known PV marker, EXP2, but it also partially overlaps with the mitochondria (Fig. 5A, top panels), suggesting that it might be localized to both subcellular organelles. GAPM1^mNG-apt^ localizes to the parasite periphery at 4 hpi, and shows colocalization with EXP2 (Fig. 5A, bottom panels). To corroborate our observations by IFAs, we used light-sheet microscopy (LSM) to determine the subcellular localization of VAC and GAPM1 with respect to EXP2 and MAHRP1 (Fig. 5A,C), which is another exported membrane protein that is trafficked to MCs (Fig. 1F). Both proteins show a high degree of colocalization with EXP2, strengthening our previous observation. Additionally, colocalization of VAC and GAPM1 was observed with respect to the exported protein MAHRP1, confirming that all three proteins are in 4-hpi parasites (Fig. 5B,D). Despite both proteins showing a high degree of colocalization with the PVM marker EXP2, our observations using structured illumination microscopy (SIM) (Fig. S4) showed a more juxtaposed, but not completely overlapping, localization. This suggests VAC and GAPM1 do not localize to the PVM but rather to a different membrane, such as the PPM.

**Fig. 6.**
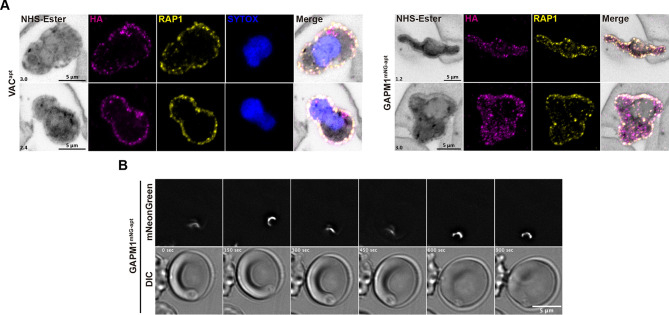
**VAC^apt^ and GAPM1^mNG-apt^ localize to the host–parasite interface.** (A) Representative images of VAC^apt^ and GAPM1^mNG-apt^ parasites showing their localization in early-ring stages (4 hpi). Tightly synchronous parasites were expanded by U-ExM, fixed with PFA, and stained with NHS-Ester (grayscale), anti-HA (magenta) and anti-RAP1 (yellow). Selected *Z*-stack images were projected as a combined single image. Number on image=Z-axis thickness of projection in µm. (B) Time course images showing GAPM1^mNG-apt^ localization after invasion of red blood cells. Images of GAPM1^mNG-apt^ from left to right are phase-contrast and mNeonGreen (white). Video captions were deconvolved and projected as a combined single image. All results are representative of three experimental repeats.

The limited resolution provided by conventional microscopy, together with the lack of available PPM markers and the narrow space between the PPM and PVM, makes it challenging to determine the precise localization of VAC and GAPM1 within the membranes. In an effort to overcome these limitations, we employed ultrastructural expansion microscopy (U-ExM) in combination with NHS-ester immunostaining, which has been demonstrated to allow visualization of various membranes, such as the nuclear membrane and the PPM (Liffner and Absalon, 2021; Liffner et al., 2023). We found that GAPM1 and VAC colocalized with the PV lumen marker, Rhoptry associated protein 1 (RAP1, Fig. 6A) (Riglar et al., 2011). Our observations indicate that VAC and GAPM1 are localized within the PVM, overlapping with the PV lumen marker RAP1 (Fig. 6A). However, based on the U-ExM data, it remains unclear whether these proteins are localized in the PPM (Fig. 6A). Interestingly, VAC exhibits an additional focal localization towards the cytoplasmic side of the parasites, which supports our previous observation suggesting a dual localization of the protein in early-stage parasites (Fig. 6A, top panels). Finally, we utilized live-cell microscopy to track the localization of mNeonGreen-tagged GAPM1 after invasion of RBCs (Fig. 6B, Movies 1 and 2). These data show that GAPM1 remains present for at least 15 min post invasion (Fig. 6B; Movies 1 and 2). Together with our previous data, this suggests that the IMC might not completely disappear until later during ring-stage development, contrary to what was previously suggested (Riglar et al., 2013). Another possibility, consistent with prior observations, is that GAPM1 relocalizes from the IMC to the PPM. Our observations cannot distinguish between these possibilities.

Together, these results show that both GAPM1 and VAC localize to the parasite periphery together with SBP1^TbID^ at the PV at 4 hpi, as suggested by the proximity-labeling data.

## DISCUSSION

The protein–protein interactions that usher exported proteins to their final destinations in the RBC via the secretory pathway are transient in nature. Previously, IP-based methods have been used to identify proteins required for the export of *P. falciparum* proteins, such as the PTEX complex (de Koning-Ward et al., 2009) and the EPIC complex (Batinovic et al., 2017). Although IP-based approaches are well-suited for identifying stable complexes, they are unlikely to identify transient interactions. A putative additional translocon at the PPM required for extracting exported membrane proteins that are inserted into the PPM during transport has long been proposed (Gruring et al., 2012; Beck and Ho, 2021; Garten and Beck, 2021). As yet, no candidates for this putative complex have been identified (Fig. 1).

In our study, we used time-resolved biotinylation to identify transient interactions of an exported membrane protein, SBP1, during its export. This approach uses a rapid and promiscuous biotin ligase to biotinylate proximal proteins (Branon et al., 2018). It is important to note that TurboID is a highly efficient enzyme and can utilize a minimal amount of biotin in the medium. Hence, to ensure that TurboID is active only at specific times we utilized biotin-free medium for this time-resolved approach to identify transient interactors of SBP1 during its trafficking in the infected RBC. As biotinylation is a permanent modification, even transient interactions can be potentially identified. Using this approach, we found putative candidates localized at the parasite periphery that could help extract membrane exported proteins from the PPM for transport into the RBC. Our data show that fusion of TurboID to the exported TM-containing protein SBP1 did not alter its trafficking to the MC nor did it have any effect on parasite growth. These data also suggest that TurboID is enzymatically active during transit in the parasite secretory pathway. This property of TurboID might be useful in many contexts to resolve protein trafficking pathways in other organisms.

A previous study on the SBP1 interactome at their final location at the MC identified 88 parasite proteins as putative interactors (Takano et al., 2019). Most of their top-ranked hit proteins were also identified in our study, such as PfEMP1, Pf332, PIESP2, REX1, MAHRP1, PTP1 and vapA. However, these were not highly enriched (≤10-fold) in the post-export interactor fraction. This could be because some of these proteins are co-transported with SBP1 and, thus, are identified in the pre-export fraction as well. Members of the PTEX complex such as EXP2, HSP101 and Trx2, were also identified in the pre-export fraction, albeit at levels below statistical significance. In addition, PTP2 and PfG174, which have been previously shown to localize as residents (Maier et al., 2008) or transient interactors (Vincensini et al., 2005) of the MCs, were more than 10-fold enriched at the post-export time point, demonstrating the reliability of our approach for identification of SBP1 interactors. Another subset of proteins identified in our study as post export interactors of SBP1 are ribosomal proteins, which have been previously observed to be exported to the *P. falciparum-*infected RBC (Das et al., 2012). Together, these data strongly suggest that the time-resolved, rapid biotinylation approach was working as designed. Given that our focus was to identify pre-export interactors, we did not pursue these proteins for further study.

Using label-free quantitative proteomics, we identified a group of 24 putative candidates that are proximal to SBP1 prior to its export to the RBC. Several of the proteins identified (14/24) were uncharacterized proteins or nuclear proteins. Because we undertook this approach to identify the putative translocon complex required for extraction of exported membrane proteins from the plasma membrane, we did not pursue the function of these proteins in this study. Translocons function to transport proteins across membranes and, therefore, we hypothesized that membrane-associated proteins in this list could putatively function as translocons. There were two putative candidates in the pre-export interactors of SBP1 that were membrane-associated – VAC and GAPM1. However, their localization in early ring-stage parasites was unknown. Therefore, to study the function of VAC and GAPM1 in early ring-stage parasites, we successfully generated conditional mutants. The data show that both VAC and GAPM1 play important functions in parasite survival within the infected RBC. Knockdown of these proteins inhibited parasite growth. However, achieving protein knockdown takes ∼24–48 h and results in parasite death prior to invasion of the RBC. Proteins transported to the MC are synthesized and transported early in the asexual lifecycle (2–8 hpi). Therefore, this prevents the characterization of their role in export, as the knockdown takes effect after proteins are exported to the MC and parasites die prior to reaching the next lifecycle. Similar to what is found for the PTEX translocon, EXP2 (Garten et al., 2018), it is likely that both GAPM1 and VAC have other essential functions in the asexual life cycle. Defining their function in export will require using a more rapid knockdown approach that has similar kinetics to that of SBP1 export, such as using degradation-domain-based tools (Beck et al., 2014; Muralidharan et al., 2012) or rapid mislocalization-based methods (Birnbaum et al., 2017).

VAC has a β-barrel porin domain that can form an aqueous channel in the membrane and function as a translocon in mitochondria and other plastids (Araiso et al., 2022). In a recent proximity-biotinylation-based proteomic screen to catalog mitochondrial proteins in *P. falciparum*, VAC was pulled down in the membrane fraction of parasite lysates, and not in the mitochondrial fraction (Araiso et al., 2022). In addition, VAC is predicted to not contain a mitochondrial targeting sequence, in contrast to its *Plasmodium* ortholog, TOM40 (PF3D7_0617000), which has a mitochondrial-targeting sequence, suggesting that VAC might not be localized to the mitochondria (Claros and Vincens, 1996). Our data reveal that VAC localizes at the host–parasite interface in early ring stages. Although there is some overlap of VAC with the mitochondria, there is a stronger overlap between the PVM marker, EXP2 and VAC in lower-resolution IFAs. It is also possible that VAC is dually localized both to the mitochondria as well as to the host–parasite interface. Superresolution microscopy suggests that EXP2 and VAC are closely juxtaposed but with minimal overlap. This suggests that VAC localizes to a compartment in close proximity to the PVM, most likely the PPM. By contrast, GAPM1 has seven TM domains and is from an apicomplexan-specific family of proteins (Bullen et al., 2009). GAPM1 has been localized to the IMC in schizont-stage parasites (Bullen et al., 2009). The IMC plays an essential role in the invasion of merozoites into the RBC; however, it is unclear what happens to the IMC proteins post invasion (Ferreira et al., 2020). Lower resolution IFAs show that GAPM1 colocalizes with the PVM-localized EXP2 in early ring-stage parasites. Using both U-ExM and SIM we observe that, like VAC, GAPM1 is in close juxtaposition with EXP2, but does not completely overlap it, suggesting that GAPM1 might also localize to the PPM in early rings. These data further suggest that the IMC could fuse to the parasite plasma membrane after merozoite invasion or that GAPM1 relocalizes to the PPM. Our data is consistent with both models and cannot distinguish between them. Cryo-EM studies of during and after parasite invasion might illuminate the fate of the IMC in newly invaded rings. Using U-ExM allowed us to observe the localization of VAC and GAPM1 at a better resolution and supports our observations that they are localized at the parasite periphery in early ring stage parasites. However, we were unable to differentiate between the PV lumen using RAP1 (Riglar et al., 2011) and the PPM, where GAPM1 and VAC might localize. Although our extensive microscopy data show that both EXP2 and RAP1 are in close proximity to both GAPM1 and VAC, neither EXP2 nor RAP1 was reproducibly identified in our mass spectrometry experiments. Together these strongly suggest the proximity labeling approach worked to identify specific interactors of SBP1 prior to its export from the parasite. These findings are consistent with the model that VAC and GAPM1 transiently interact with SBP1 prior to its export at the parasite periphery, perhaps as members of a putative translocon complex.

Several mechanistic aspects of this model remain to be resolved but, similar to the PTEX complex, which was first identified as a putative complex at the PV membrane (de Koning-Ward et al., 2009), both VAC and GAPM1 are at the right place at the right time. Furthermore, VAC has a porin translocon domain, which could function in a manner analogous to the mitochondrial outer membrane translocon to extract membrane-anchored exported proteins from the parasite plasma membrane. This hints that this ancient porin domain protein has been repurposed by *Plasmodium* parasites on the PPM to facilitate export of membrane proteins to the infected RBC.

## MATERIALS AND METHODS

### Construction of SBP1 plasmids

Genomic DNA was isolated from *P. falciparum* NF54^attB^ cultures using the QIAamp DNA blood kit (Qiagen). PCR products were inserted into the respective plasmids using ligation-independent cloning (SLIC), as described previously (Cobb et al., 2017), or the NEBuilder HiFi DNA Assembly system (NEB). All constructs used in this study were confirmed by sequencing. All primers used in this study are in Table S3.

For generation of the plasmid pTOPO-SBP1-TbID, sequences of ∼500 bp of homology to the SBP1 C-terminus and 3′UTR were amplified using primer pairs P1–P2 and P3–P4, respectively, and the sequence of V5-tagged TurboID was amplified using primers P5 and P6 (Table S3). For expression of a SBP1 gRNA, oligonucleotides P17–P18 were inserted into cut pUF1-Cas9 (Table S3).

For generation of the plasmid pKD-VAC-Apt, sequences of ∼450 bp of homology to the Pf1432100 C-terminus and 3′UTR were amplified using primer pairs P7–P8 and P9–P10, respectively (Table S3). Amplicons were then inserted into pKD (Cobb et al., 2017; Rajaram et al., 2020) digested with AatII and AscI. For expression of a Pf1432100 gRNA, oligonucleotide P19 was inserted into cut PUF1-Cas9 (Table S3).

For generation of the plasmid pKD-GAPM1-mNG-Apt, sequences of ∼500 bp of homology to the PfGAPM1 C-terminus and 3′UTR were amplified using primer pairs P11–P12 and P13–P14, respectively, and the sequence of mNeonGreen was amplified using primers P15 and P16 (Table S3). Amplicons were then inserted into pKD (Rajaram et al., 2020) digested with AatII and AscI. For expression of PfGAPM1 gRNA, oligonucleotide P20 was inserted into cut PUF1-Cas9 (Table S3).

### Parasite culture and transfections

*Plasmodium* parasites (Nkrumah et al., 2006) were cultured in RPMI 1640 medium (NF54^attB^, VAC^apt^ and GAPM1^mNG-apt^) or in biotin-free medium (SBP1^TbID^) (Zimbres et al., 2020) supplemented with AlbuMAX I (Gibco), and transfected as described previously (Kudyba et al., 2018).

For generation of SBP1^TbID^ parasites, a mix of two plasmids (50 µg each) were transfected into NF54^attB^ parasites in duplicate. The plasmid mix contained the plasmid pUF1-Cas9-SBP1gRNA, which contains the DHOD resistance gene and the marker-free plasmid pTOPO-SBP1-TbID. Drug pressure was applied 48 h after transfection, using 1 µM DSM1 (Ganesan et al., 2011) and selecting for Cas9 expression. After parasites grew back from transfection, integration was confirmed by PCR, and then cloned using limiting dilution. After clonal selection, cultures were transferred to biotin-free medium without DSM1.

For generation of VAC^apt^ and GAPM1^mNG-apt^ parasites, the pKD-VAC-Apt and pKD-GAPM1-mNG-Apt plasmids (20 µg) and the respective pUF1-Cas9 plasmid (50 µg) were transfected into NF54^attB^ parasites in duplicate. Before transfection pKD plasmids were digested overnight with EcoRV (NEB). The enzyme was then subjected to heat inactivation for 20 min at 65°C and then mixed with the pUF1-Cas9 plasmid. Transfected parasites were grown in 0.5 µM anhydrous tetracycline (aTc) (Cayman Chemical). Drug pressure was applied 48 h after transfection, using blasticidin (BSD; Gibco A1113903) at a concentration of 2.5 µg/ml, selecting for pKD-VAC-Apt and pKD-GAPM1-mNG-Apt expression. After parasites grew back from transfection, integration was confirmed by PCR, and then cloned using limiting dilution. Clones were maintained in mediums containing 0.5 µM aTc and 2.5 µg/mL BSD.

### Growth assays

For all assays, aliquots of parasite cultures were incubated in 8 µM Hoechst 33342 (Thermo Fisher Scientific) for 20 min at room temperature and then fluorescence was measured using a CytoFlex S (Beckman Coulter) flow-cytometer. Flow cytometry data were analyzed using FlowJo software (Tree Star, Inc.) and plotted using Prism (GraphPad Software, Inc.).

For the SBP1^TbID^ growth assay, asynchronous parasites were transferred to a 96-well plate at 0.5% parasitemia and grown for 4 days. Parasitemia was monitored every 24 h.

For the VAC^apt^ and GAPM1^mNG-apt^ growth assays, synchronous ring-stage parasites were washed five times with RPMI 1640 medium and split into two cultures, one resuspended in medium containing 0.5 µM aTc and 2.5 µg/ml BSD, and the other one in medium containing only 2.5 µg/ml BSD. Then cultures were transferred to a 96-well plate at 0.2% parasitemia and grown for 6 days. Parasitemia was monitored every 48 h.

### Western blotting

For SBP1^TbID^ parasites, RIPA buffer (150 mM NaCl, 20 mM Tris-HCl pH 7.5, 1 mM EDTA, 1% SDS and 0.1% Triton X-100) and sonication was used to lyse parasite pellets conserving all exported proteins. Briefly, late-stage parasites were first isolated using a Percoll gradient (Genesee Scientific). The resulting pellets were then resuspended in RIPA buffer and sonicated three times at 20% amplitude for 20 s each. Protein supernatants were solubilized in protein loading dye with β-mercaptoethanol (LI-COR Biosciences) and used for SDS-PAGE.

For VAC^apt^ and GAPM1^mNG-apt^ parasites, ice-cold 0.04% saponin in 1× PBS was used to isolate parasites from host cells. The parasite pellets were subsequently solubilized in protein loading dye with β-mercaptoethanol (LI-COR Biosciences) and used for SDS-PAGE.

Primary antibodies used in this study included: mouse-anti-V5 (cat. no 80076, Cell Signaling Technology, 1:1000), rabbit-anti-PfEF1α (from Dan Goldberg, Depts of Medicine and Molecular Microbiology, Washington University School of Medicine, St Louis, MO 63110, USA; 1:2000), and mouse-anti-HA 6E2 (cat. no 2367, Cell Signaling Technology, 1:2000). Secondary antibodies used were IRDye 680 CW goat-anti-rabbit IgG, IRDye 800CW goat-anti-mouse IgG, and IRDye 800CW Streptavidin (LI-COR Biosciences, 1:20,000 and 1:10,000). Membranes were imaged using the Odyssey Clx LI-COR infrared imaging system (LI-COR Biosciences). Images were processed and analyzed using ImageStudio (LI-COR Biosciences). Uncropped images of blots presented in this study are shown in Fig. S5.

### Immunofluorescence microscopy

For IFAs, cells were fixed following the previously described protocol (Cobb et al., 2017). The SBP1^TbID^ cell line was smeared onto a slide and fixed with acetone. The VAC^apt^ and GAPM1^apt^ cell lines were fixed with 4% paraformaldehyde (PFA; Electron Microscopy Sciences) and 0.03% glutaraldehyde.

Primary antibodies used in the IFAs included mouse-anti-V5 TCM5 (cat. no 12-6796-42, eBioscience, 1:100), rabbit-anti-V5 D3H8Q (cat. no 13202, Cell Signaling Technology, 1:100), rabbit-anti-HA SG77 (cat. no 71-5500, Thermo Fisher Scientific, 1:100), rabbit-anti-MAHRP (from Hans-Peter Beck, Department of Medical Parasitology and Infection Biology, Swiss Tropical Institute, Allschwil, Switzerland; 1:500), mouse-anti-EXP2 7.7 and mouse-anti-KAHRP (from Graeme Cowan, The European Malaria Reagent Repository, www.malariaresearch.eu; 1:1000, 1:500, respectively). Secondary antibodies used were conjugated to Alexa Fluor 488, Alexa Fluor 546, and streptavidin Alexa Fluor 488 (Life Technologies, 1:1000).

After mounting the cells using ProLong Diamond with DAPI (Invitrogen), they were imaged using a DeltaVision II microscope system with an Olympus Ix-71 inverted microscope. Images were collected as a *Z*-stack and deconvolved using SoftWorx (GE Healthcare), then displayed as a maximum intensity projection. Adjustments to brightness and contrast were made for display purposes using Adobe Photoshop.

### Synchronization assays

To detect SBP1 during export, SBP1^TbID^ parasites were synchronized using two rounds of 5% sorbitol treatment. Subsequently, schizont-stage parasites were isolated using a Percoll gradient (Genesee Scientific) and promptly transferred to freshly pre-warmed fresh RBCs at a hematocrit of 1% (Interstate Blood Bank Inc, Memphis, TN 38104, USA). Parasites were then allowed to undergo egress and invade new RBCs, and samples were collected at various time points for IFAs.

### SBP1^TbID^ proximity biotinylation and mass spectrometry

To confirm the biotinylation of proteins by TbID-tagged SBP1, SBP1^TbID^ parasites were collected for western blotting and IFAs after a 2-h incubation in biotin-free medium supplemented with 50 µM biotin (Cambridge Isotope Laboratories Inc., catalog no. ULM-3129-0.005).

For the detection of SBP1 during export, SBP1^TbID^ parasites were synchronized with two series of 5% sorbitol treatment. Late-schizont-stage parasites were isolated by performing a Percoll (Genesee Scientific) gradient separation. The isolated parasites were then split into two samples and immediately transferred to RBCs at a hematocrit of 1% in warm medium, one sample without biotin and the other group with biotin (50 µM). Both parasite cultures were incubated for 4 h at 37°C with shaking to allow egress and invasion of new RBCs. Afterward, cultures were treated with 5% sorbitol to remove any remaining late-stage parasites.

The biotinylated culture was washed with 1× PBS, incubated on ice for 10 min to inactivate the biotinylation process and then stored at −80°C until further processing. The non-biotinylated culture was incubated for 16 h at 37°C with shaking, followed by a 4-h incubation in medium containing biotin. Finally, the parasites were collected as described above.

To isolate biotinylated proteins, parasite pellets were lysed using an extraction buffer containing 40 mM Tris-HCl pH 7.6, 150 mM KCl, 1 mM EDTA, 5% NP-40 and 1× HALT protease inhibitor cocktail (Thermo Fisher Scientific, cat. no. 78438). Sonication was performed three times at 10% amplitude with 20-s pulses. Streptavidin MagneSphere paramagnetic particle beads (Promega) were used to capture biotinylated proteins. The beads were washed three times in 1 ml of 1× PBS. Protein lysates were then incubated with the streptavidin beads for 1 h at room temperature. After removing the unbound fraction, the magnetic beads were washed twice with an extraction buffer and once with 1× PBS. The biotinylated proteins bound to the magnetic beads were digested and analyzed at the Proteomics and Metabolomics shared resource at Fred Hutchinson Cancer Research Center using a Orbitrap Fusion with ETD Mass Spectrometer. The mass spectrometry proteomic data have been deposited to the ProteomeXchange consortium via the MassIVE partner repository with the dataset identified PXD034946.

### U-ExM

Cultures for U-ExM were synchronized to 4-h rings, following the previously described synchronization method. Ultrastructure expansion microscopy (U-ExM) was performed as described previously (Liffner and Absalon, 2021), with minor modifications.

To start, 12 mm round coverslips were treated with poly-D-lysine for 1 h at 37°C. They were then washed three times with MilliQ water and placed in a 24-well plate. Parasite cultures with ∼5% parasitemia were adjusted to 0.5% hematocrit. Then, 1 ml of parasite culture was added to the well containing the treated coverslip and incubated for 1 h at 37°C.

After the incubation, the supernatant was carefully removed, and a fixative solution (4% v/v PFA in PBS) was added, followed by a 20 min incubation at 37°C. The coverslips were washed three times with 1× PBS and incubated overnight at 37°C in 500 µl of 1.4% formaldehyde and 2% acrylamide (FA/AA) in PBS.

The monomer solution (19% sodium acrylate, 10% acrylamide and 0.1% N,N′-methylenebisacrylamide in PBS) was prepared a day prior to use and stored at −20°C. Before gelation, coverslips were removed from FA/AA solution and washed three times in 1× PBS.

For gelation, 5 µl of 10% tetramethylenediamine (TEMED) and 5 µl of 10% ammonium persulfate (APS) were added to 90 µl of the monomer solution, briefly vortexed and 35 µl of the monomer mixture were pipetted onto parafilm. The coverslips were placed on top with the cell side facing down, and the gels were incubated at 37°C for 30 min.

Next, the gels were transferred into a 6-well plate containing denaturing buffer (200 mM SDS, 200 mM NaCl, 50 mM Tris-HCl, pH 9) and incubated for 15 min incubation at room temperature. Afterward, the gels were separated from the coverslips and transferred to 1.5 ml tubes with the denaturing buffer for 90-min incubation at 95°C.

Subsequently, the gels were incubated with secondary antibodies diluted in 1× PBS for 2.5 h. After denaturation, gels were transferred to Petri dishes containing 25 ml of MilliQ water and incubated three times for 30 min at room temperature with shaking, changing the water in between. The gels were measured and subsequently shrunk using two washes with 1× PBS. They were then transferred to a 24-well plate for blocking in 3% BSA in PBS at room temperature for 30 min. After blocking gels were incubated with primary antibodies diluted in 3% BSA overnight at room temperature.

Following primary antibody incubation, the gels were washed three times in 0.5% PBS with 0.1% Tween 20 for 10 min before incubation with secondary antibodies diluted in 1× PBS for 2.5 h.

After secondary antibody staining, the gels were washed three times with 0.5% PBS with 0.1% Tween 20. Then, gels were transferred back to 10 cm Petri dishes for the second round of expansion, involving three incubations with MilliQ water. After re-expansion, the gels were either imaged immediately or stored in 0.2% propyl gallate in water until imaging.

The primary antibodies used were: rat-anti-HA 3F10 (cat. no 12158167001, Roche, 1:50) and mouse-anti-RAP1 2.29 (Graeme Cowan, 1:500; Hall et al., 1983). The secondary antibodies used were conjugated to Alexa Fluor 488 and Alexa Fluor 546 (Life Technologies, 1:500), NHS-ester 405 (Thermo Fisher Scientific, 1:250). The gels were imaged using a Zeiss LSM 980 microscope with Airyscan 2. Images were collected as a *Z*-stack, processed by Airyscan, and then displayed as a maximum intensity projection. Adjustments to brightness and contrast were made using ZEN Blue software for display purposes.

### Live microscopy

Parasites were initially synchronized using a Percoll gradient and 5% sorbitol treatment. Schizont-stage parasites were then enriched using magnetic separation with LD columns (MACS, Miltenyi Biotec). Subsequently, the enriched parasites were incubated for 4 h at 37°C in pre-warmed RPMI medium supplemented with 25 nM ML10 compound (BEI Resources, catalog no. NR-56525, www.beiresources.org) as described previously (Ressurreição et al., 2020).

After the 4-h incubation, parasites were washed once with pre-warmed RPMI medium and immediately transferred to pre-warmed fresh RBCs at a hematocrit of 0.25%.

For live-cell imaging, a sample of the parasite culture was transferred to a 35 mm glass bottom dish at 1.5 h post wash, and observed using a DeltaVision II microscope system with an Olympus Ix-71 inverted microscope. The imaging process involved capturing images for 15 min with a frame interval of 15 s, starting from the observation of parasite invasion. Imaging was conducted within environmental chambers set at 37°C and 5% CO2. Images and videos were processed using the FIJI software.

## Supplementary Material

10.1242/joces.260506_sup1 Supplementary information

Table S1. Complete list of proteins identified using label-free analysis and collected by mass spectrometry.
