# Supplementary material for: Time-resolved proximity biotinylation implicates a porin protein in export of transmembrane malaria parasite effectors
Source: J Cell Sci. 2023 Oct 18;136(20):jcs260506. doi: 10.1242/jcs.260506 (PMC10651097; doi:10.1242/jcs.260506)
Supplement: Supplementary information [file joces-136-260506-s1.pdf]

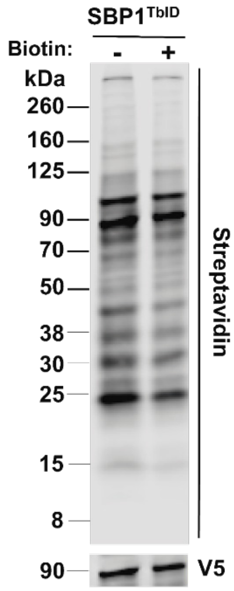

Fig. S1. Biotinylation of proximal proteins by TurboID$_{V5}$-tagged SBP1. Western blot of parasite lysates isolated from the mutant line SBP1^{TbID} grown in complete RPMI medium, incubated with or without biotin (50 μM) for 2 h. Samples were probed with antibodies against V5 (loading control) and fluorescent dye-labeled streptavidin. The protein marker sizes are shown on the left.

Journal of Cell Science • Supplementary information

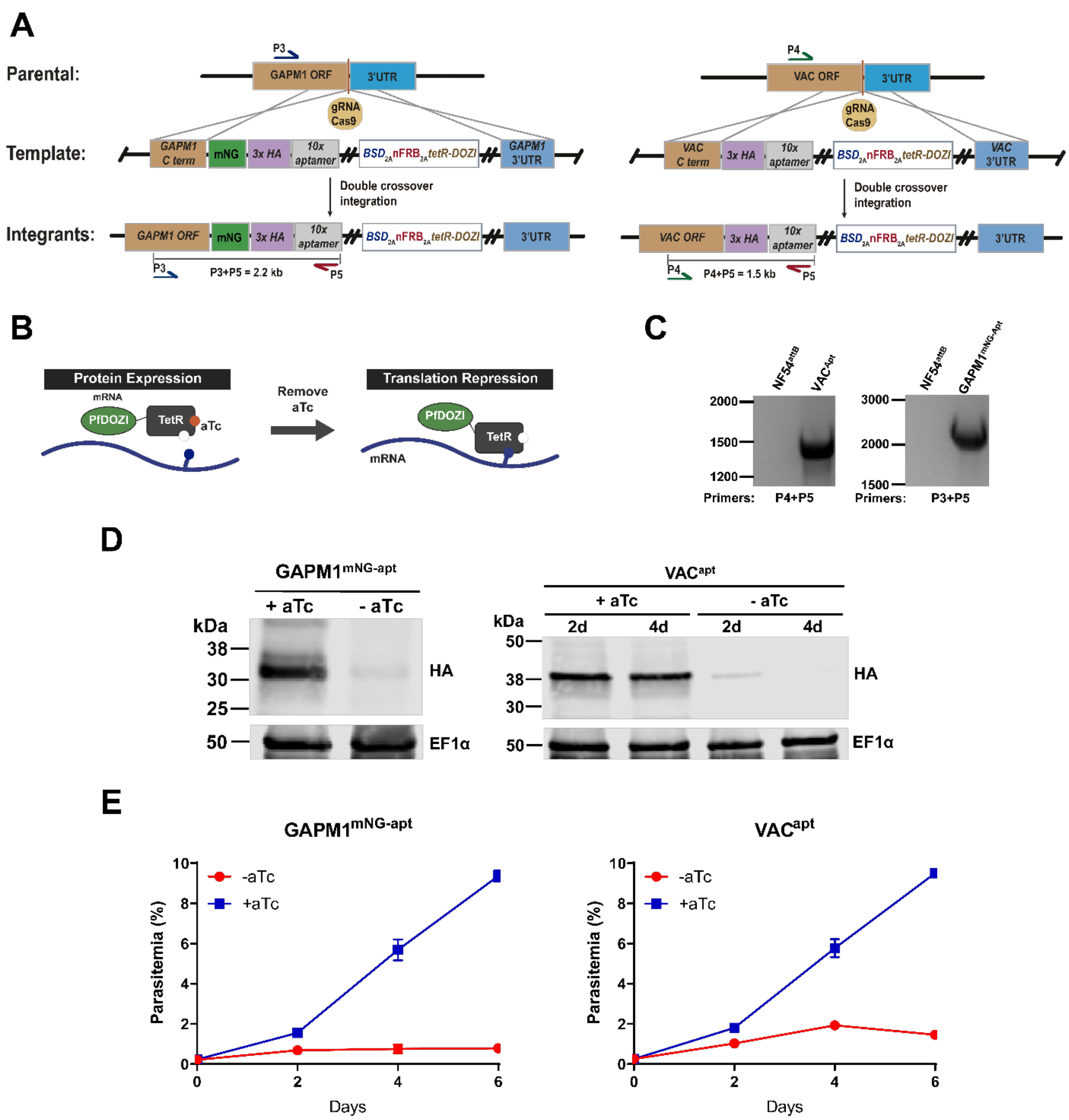

Fig. S2. Characterization of parasite lines VAC^{apt} and GAPM1$^{mNG\text{-}apt}$. (A) Schematic showing the integration of the repair plasmid to modify the genomic loci of Pf3D7_1432100 (VAC) and Pf3D7_1323700 (GAPM1). Cas9 introduces a double-stranded break at the C-terminus of the VAC and GAPM1 locus. The repair plasmid provides homology regions for double-crossover homologous recombination, introducing the HA-tag and the TetR-Aptamer system. For GAPM1$^{mNG\text{-}apt}$, a fluorescent tag mNeonGreen was introduced between the C-terminus and the HA-tag. (B) Regulation of protein expression using the TetR-Aptamer knockdown system. TetR binds to aptamer repeats in the mRNA, while PfDOZI localizes the complex to sites of mRNA sequestration, causing a repression in translation of the gene of interest. Anhydrous tetracycline (aTc) binds to TetR, blocking its interaction with the aptamers. (A) PCR test confirming integration at the VAC and GAPM1 locus. Amplicons were amplified

from genomic DNA isolated from mutant and wild-type parasites. Primers were designed to amplify the region between the C-terminus and the tandem of 10X aptamer repeats. (B) Western blot of parasite lysates isolated from the mutant lines VAC^{apt} and $GAPM1^{mNG\text{-}apt}$ probed with antibodies against HA and EF1α (loading control). The protein marker sizes are shown on the left. $GAPM1^{mNG\text{-}apt}$ parasites were collected after incubation for 48 h in the presence or absence of aTc. VAC^{apt} parasites were collected after incubation for 48 and 96 h in presence or absence of aTc. (C) Growth of synchronous VAC^{apt} and $GAPM1^{mNG\text{-}apt}$ parasites over 6 days after removal of aTc from the medium via flow cytometry. Representative of three biological replicates shown for each growth curve. Each data point represents the mean of three technical replicates; error bars represent standard deviation.

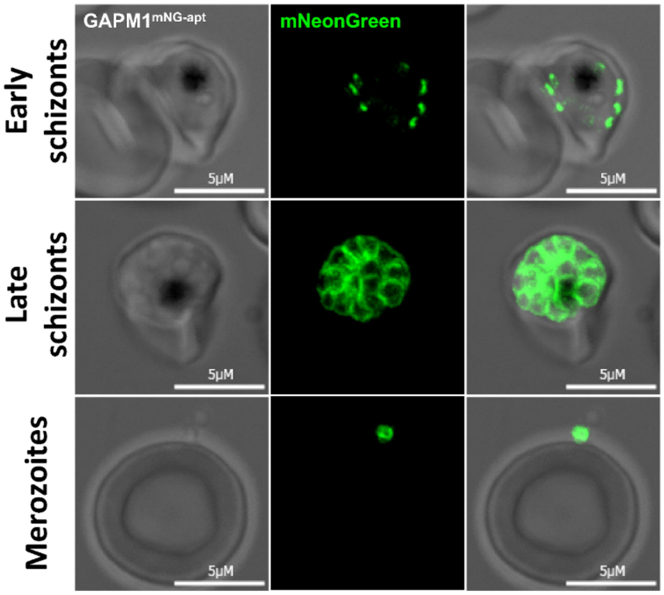

Fig. S3. Localization of GAPM1 at late stages in the GAPM1$^{mNG\text{-}apt}$ cell line. (A) Representative live images showing GAPM1$^{mNG\text{-}apt}$ localization at early and late schizonts, and merozoites. Images of GAPM1$^{mNG\text{-}apt}$ from left to right are phase-contrast, mNeonGreen (green), and fluorescence merge. Z stack images were deconvolved and projected as a combined single image.

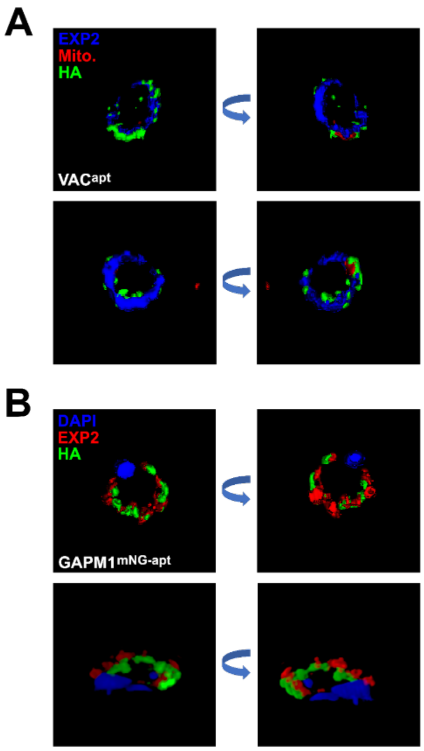

Fig. S4. Localization of VAC and GAPM1 at early-stage parasites. 3D reconstruction based on structured illumination microscopy images captured from (A) VAC^{apt} and (B) GAPM1$^{mNG-apt}$ ring-stage parasites at 4 hpi and stained with the antibodies as in Fig 5A.

Figure 1, panel D:

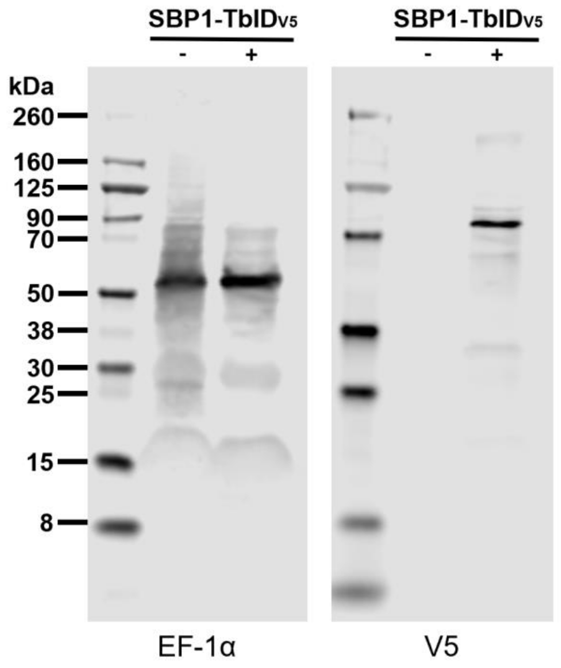

Figure 2, panel A:

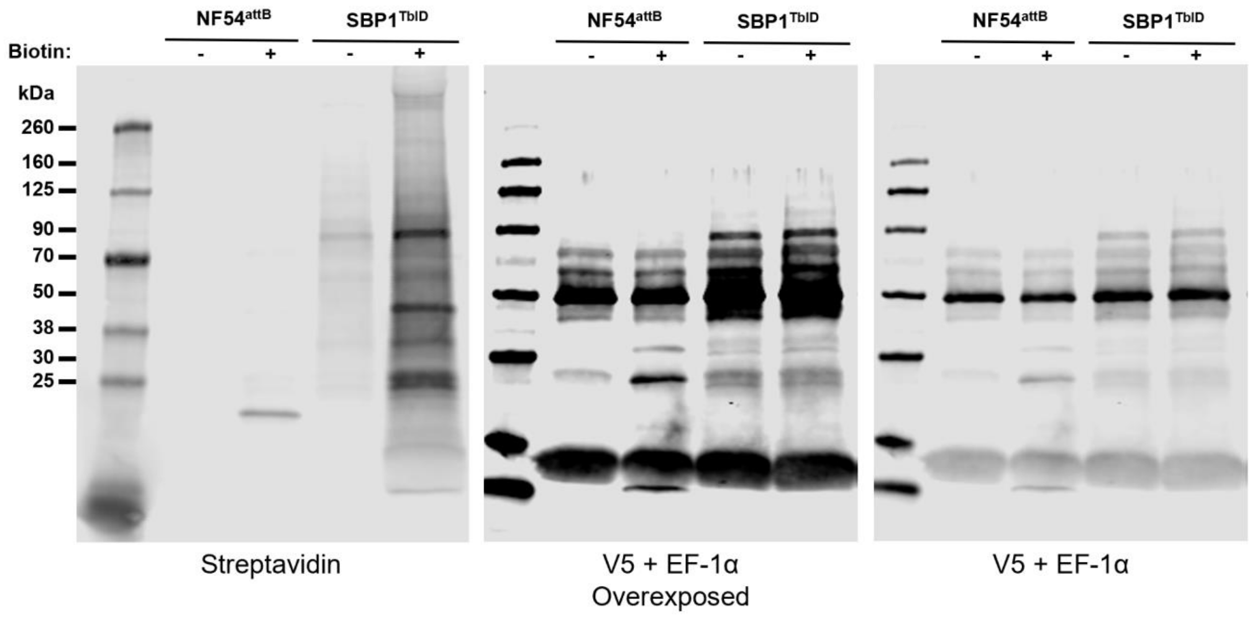

Journal of Cell Science • Supplementary information

Figure 2, panel B:

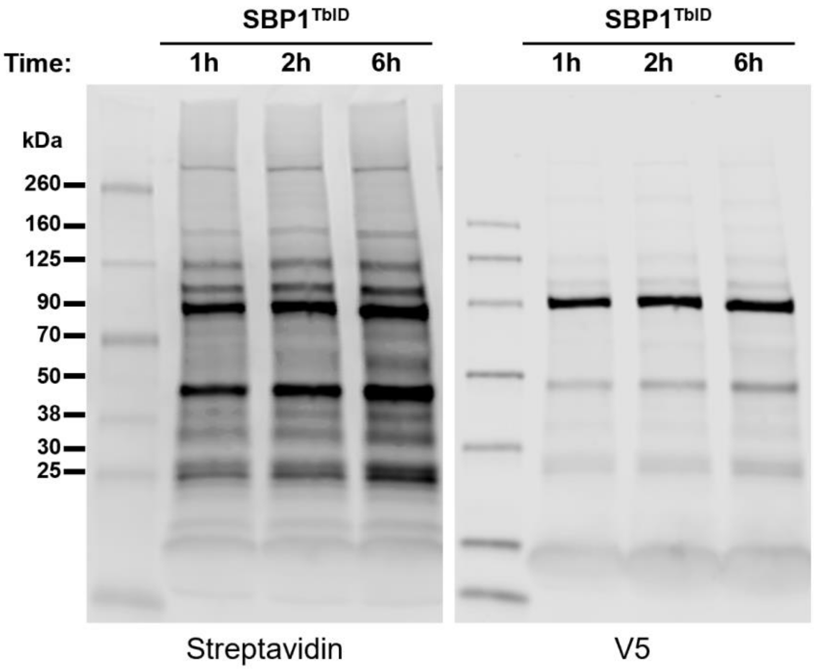

Figure 2, panel C:

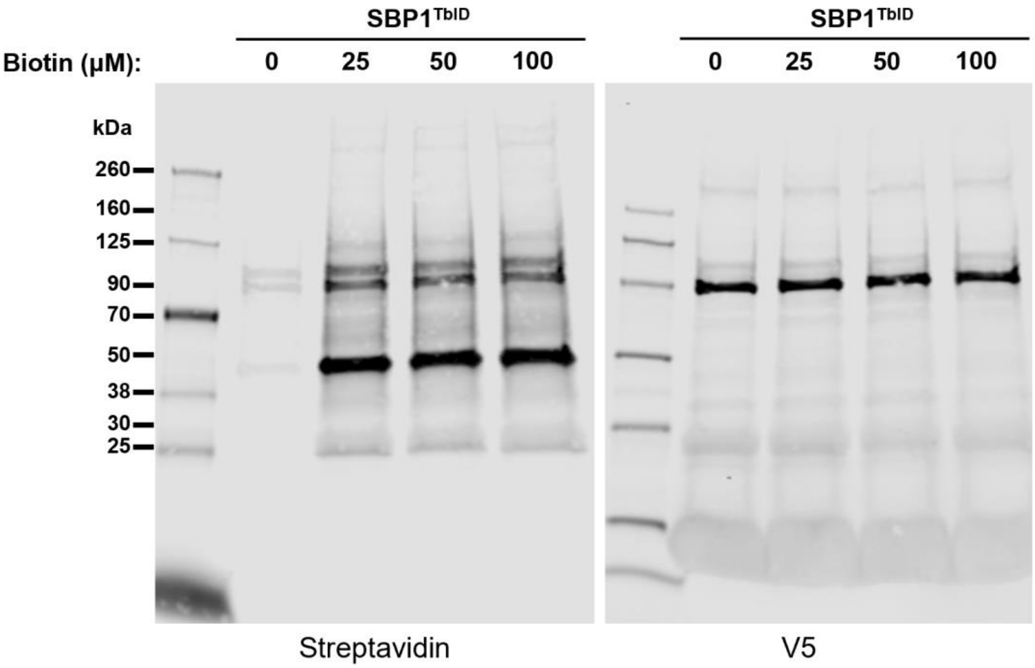

Fig. S5. Uncropped blots

Journal of Cell Science • Supplementary information

Table S1. Complete list of proteins identified using label-free analysis and collected by mass spectrometry.

Available for download at
https://journals.biologists.com/jcs/article-lookup/doi/10.1242/jcs.260506#supplementary-data

Table S2. List of putative interactors of SBP1^{TbID} at 4hpi with their homology in other Apicomplexans and their identified domains.

Gene ID	Only in P. falciparum	Other Plasmodium sp	Toxoplasma	Cryptosporidium	In Eukaryotes	Domains
PF3D7_0208100		X	X		X	C2 domain
PF3D7_0815800		X	X	X	X	VPS9 domain (RABX5)
PF3D7_1432100		X				Porin domain
PF3D7_1401600	X					PHIST domain
PF3D7_0215700		X	X	X	X	RNA polymerase RPB2 domain
PF3D7_1227600		X				Trimeric LpxA-like domain
PF3D7_0803400		X	X	X	X	SNF2-like, N-terminal domain
PF3D7_0817700		X	X			--
PF3D7_1332200		X	X	X		--
PF3D7_1323700		X	X	X		DUF3273 (Not known function)
PF3D7_0304100		X	X	X		Inner membrane domain
PF3D7_1324800		X	X		X	Mur-like catalytic domain
PF3D7_0705100		X				--
PF3D7_0321100		X				Leucine-rich repeat domain
PF3D7_0308700		X				--
PF3D7_1026600	X					--
PF3D7_0723800		X				Serralyisin-like metalloprotease
PF3D7_0308100		X	X	X	X	B-box-type zinc finger domain
PF3D7_0704300		X				--
PF3D7_0612200		X				Leucine-rich repeat domain
PF3D7_1138000		X				Sox, C-terminal
PF3D7_1239800		X				--
PF3D7_1025900		X				--
PF3D7_1014900		X				--

Journal of Cell Science • Supplementary information

Table S3. List of primers used in the study to generate the cell lines SBP1^{TbID}, VAC^{apt} and GAPM1$^{mNG\text{-}apt}$.

Primer	Amplicon	Sequence (5' – 3')
P1	SBP1-Cterm	CCCTCACTAAAGGGACTAGTCTTTGTTATTAACATATTATTGTTCATCAACTTTTACAAC
P2	SBP1-Cterm	TAGATCTGTTAACGGATCCGGTTTCTCTAGCAACTGTTTTTGTCGTTGATTTGGTTGTGG
P3	SBP1-3'UTR	TGGACAGCACCTAAGAATTCAGATAAATTATTATAAATCAATTGTGCCAACAATAATGAG
P4	SBP1-3'UTR	TAGCGGCCGCGAATTCGTTGTGAACGTTTTTAATTATGTATGCATACAAAAAATATAC
P5	TurboID	CCCTCACTAAAGGGACTAGTGCTCGGGATCCACCGGTCGCCACCATG
P6	TurboID	TAGCGGCCGCGAATTCTTAGGTGCTGTCCAGGCCCAGCAGGGGGTTG
P7	VAC-Cterm	TGCAGAAAGGTGTGGATATCATCCCGAGTAATAAACACTTTTATGGATCC
P8	VAC-Cterm	CGTCATAAGGGTATCCGGAGACGTCTGATTTTAAATAAAGTTTCATTCCAAATTTGGTG
P9	VAC-3'UTR	TCCAATGGCCCCTTTCCGGGCGCGCCTCTTATTTGTTTTTATTTATTAAGGAAGAATTAG
P10	VAC-3'UTR	TTATTACTCGGGATGATATCCACACCTTTCTGCACCTTATATATAC
P11	GAPM1-Cterm	TGGTGCTAGGTAGGGATATCGAACTGTATCATGGAGCTTGTCCCTTATATGTTTG
P12	GAPM1-Cterm	AAACGGTGGCGACCGGTGGATCCCGAGCACATTGTTTGCATGCTGCAATATTTTCGGTAG
P13	GAPM1-3'UTR	TCCAATGGCCCCTTTCCGGGCGCGCCCCTACAAATTAACAAATTCGAAGAATACAAAAG
P14	GAPM1-3'UTR	CCATGATACAGTTCGATATCCCTACCTAGCACCACATTTTAACATTG
P15	mNeonGreen	ATGTGCTCGGGATCCACCGGTCGCCACCGTTTCTAAGGGTGAAGAAGATAACATGGCTTC
P16	mNeonGreen	CGTCATAAGGGTATCCGGAGACGTCCTTGTATAATTCATCCATACCCATAACATCAGTG
P17	SBP1 gRNA	TAAGTATATAATATTTCTAGCAACTGTTTTTGTTGGTTTTAGAGCTAGAA
P18	SBP1 gRNA	TTCTAGCTCTAAAACCAACAAAAACAGTTGCTAGAAATATTATATACTTA
P19	VAC gRNA	CATATTAAGTATATAATATTTACTGTCTATAATTAAACAAGTTTTAGAGCTAGAAATAGC
P20	GAPM1 gRNA	CATATTAAGTATATAATATTATGCAAACAATGTTAAAAAGGTTTTAGAGCTAGAAATAGC

Journal of Cell Science • Supplementary information

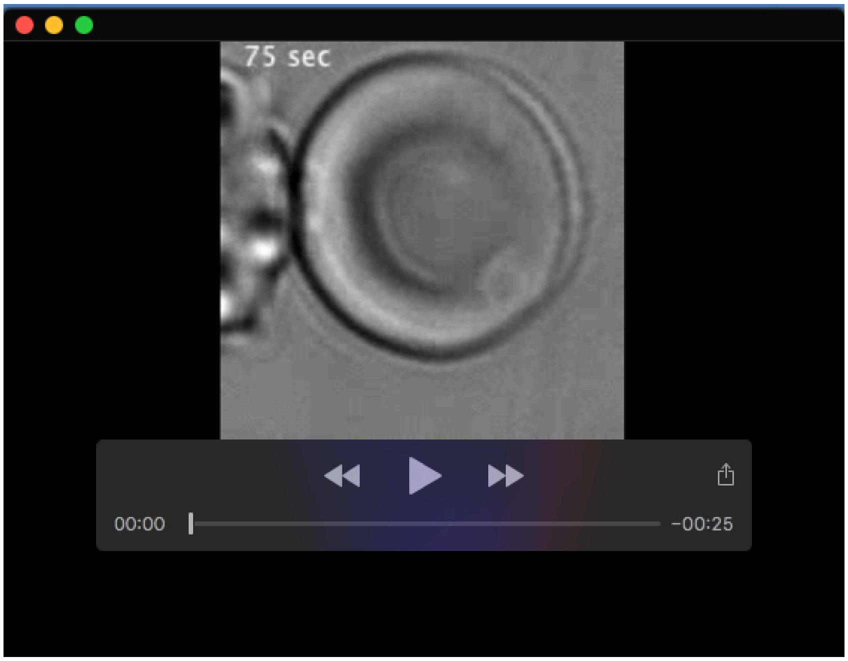

Movie 1. Live imaging of GAPM1$^{mNG\text{-}apt}$ parasites post-invasion. Phase contrast time course of 15 minutes showing a GAPM1$^{mNG\text{-}apt}$ parasite within recently invaded red blood cells. Time shown in seconds.

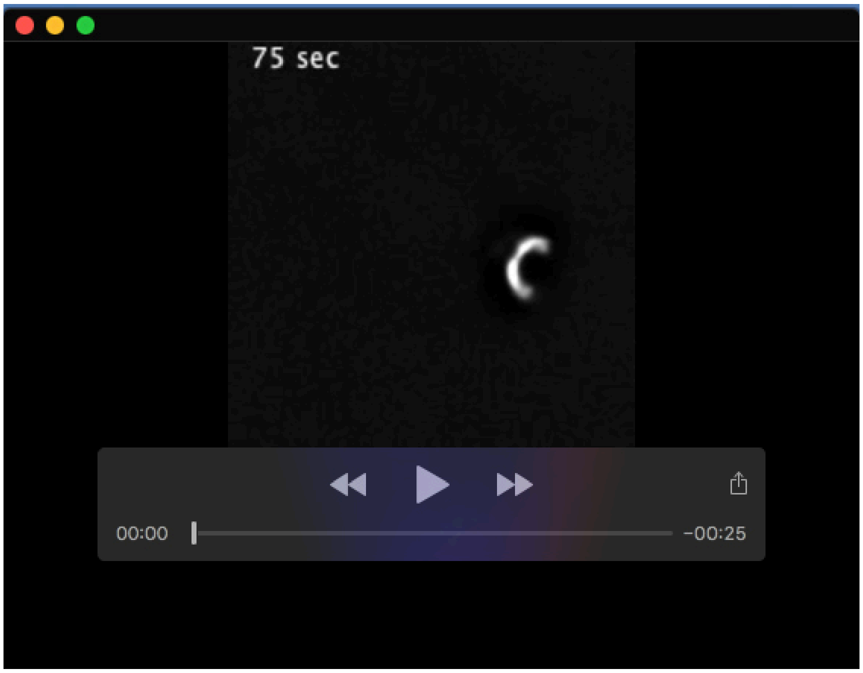

Movie 2. Live imaging of GAPM1$^{mNG\text{-}apt}$ parasites post-invasion. Fluorescence time course of 15 minutes showing a GAPM1$^{mNG\text{-}apt}$ parasite within recently invaded red blood cells. GAPM1 (white) is shown as a dynamic protein within the first hours after invasion. Time shown in seconds.
