# Supplementary material for: Time-resolved proximity biotinylation implicates a porin protein in export of transmembrane malaria parasite effectors
Source: J Cell Sci. 2023 Oct 18;136(20):jcs260506. doi: 10.1242/jcs.260506 (PMC10651097; doi:10.1242/jcs.260506)
Supplement: Supplementary file 1 [file joces-136-260506_review_history.pdf]

Time-resolved proximity biotinylation implicates a porin protein in export of transmembrane malaria parasite effectors

David Anaguano, Watcharatip Dedkhad, Carrie F. Brooks, David W. Cobb and Vasant Muralidharan
DOI: 10.1242/jcs.260506

Editor: Jennifer Lippincott-Schwartz

Review timeline

Original submission:	18 August 2022
Editorial decision:	17 October 2022
First revision received:	10 August 2023
Editorial decision:	7 September 2023
Second revision received:	16 September 2023
Accepted:	21 September 2023

Original submission

First Decision Letter

MS ID#: JOCES/2022/260506

MS TITLE: Time-resolved proximity biotinylation implicates a porin domain protein in export of malaria parasite effectors

AUTHORS: David Anaguano, Carrie F Brooks, David W Cobb, and Vasant Muralidharan
ARTICLE TYPE: Research Article

We have now reached a decision on the above manuscript.

To see the reviewers' reports and a copy of this decision letter, please go to: https://submit-jcs.biologists.org and click on the 'Manuscripts with Decisions' queue in the Author Area. (Corresponding author only has access to reviews.)

As you will see, the reviewers raise a number of substantial criticisms that prevent me from accepting the paper at this stage. They suggest, however, that a revised version might prove acceptable, if you can address their concerns. If you think that you can deal satisfactorily with the criticisms on revision, I would be pleased to see a revised manuscript. We would then return it to the reviewers.

Reviewer 1

Advance summary and potential significance to field

Summary In this manuscript the authors pursue the so-called PTEM in P. falciparum by a novel and innovative time resolved, quantitative TurboID approach. The method clearly is time-resolved, but it is not clear whether meaningful interactions toward the elusive PTEM are identified as validations of two putative candidates, which are fairly long-shots at best, is inconclusive. To come up with a mechanistic model the authors need transition of proteins from the IMC to the PPM, which seems to be supported by the data. This by itself would be a mechanistic breakthrough that would be inconsistent with what is known about IMC disassembly, which is actually very little for the RBC

© 2023. Published by The Company of Biologists under the terms of the Creative Commons Attribution License (https://creativecommons.org/licenses/by/4.0/).

stage. However, support for that is only partial at best. The VAC candidate suffers similar shortcomings.

Comments for the author

Specific points
1. Line 332 (and 438/9, 444). “we reasoned that the IMC most likely fuses to the PPM shortly after invasion”. This is a pretty bold assumption without any solid basis. At least in the Plasmodium sporozoite (Coppens lab and Meis et al in the 1980s: IMC detached from the plasma membrane accumulates in the cytoplasm) and Toxoplasma (various labs: very localized breakdown in apical to basal direction) some insights have been generated in how organized and coordinated these processes are, at least by electron microscopy. Admittedly, no mechanistic details are known, but assuming PPM fusion would dramatically expand the parasite within minutes post invasion, which does not seem to be consistent with observations made in merozoites (Gilberger lab: 15 min after completing invasion only small IMC membrane pieces remain (Riglar et al., 2013). Indeed, the presented data seem to hint at (partial or selective) fusion of IMC and PPM to some extend (or translocation of some IMC proteins?), but even that would need to be further validated.
2. The presented data do not permit to associate any of the identified candidates directly with the elusive PTEM. The presented hypothetical scenarios are tantalizing, but require a lot of assumptions and handwaving, and certainly the suggested DD or other faster kinetic protein knock-down would be needed to support these scenarios

Minor points
1. Line 239: Fig. 3C should be 3B
2. Lines 256, 263. The “square” referred to is a rectangle. “box” might be an alternative term that is more astute.

Reviewer 2

Advance summary and potential significance to field

Review of "Time resolved proximity biotinylation implicates a porin domain protein in export of malaria parasite effectors"

My recommendation is acceptance of the paper with one major and minor revisions.

Despite recent advances in vaccine development, malaria remains a worldwide public health issue with the rapid emergence of resistance to the frontline artemisinin combination therapy. In addition, clinical symptoms of malaria begin with the invasion and proliferation of Plasmodium parasites in red blood cells.

Therefore a better understanding at the molecular level of the parasitic biological processes required for parasite invasion, growth, and egress from erythrocytes will likely facilitate the development of novel antimalarial drugs.

One attractive target is to disrupt the parasite protein export pathway. To proliferate, Plasmodium falciparum, the deadliest causing agent of malaria, must remodel the erythrocytic plasma membrane through the export of hundreds of proteins across the parasitophorous vacuole membrane using the Plasmodium translocon of exported proteins or PTEX. However, how membrane proteins are extracted from the parasite plasma membrane and delivered to the PTEX complex remains poorly understood at the molecular level.

In this study, the authors took a proximal labeling-based approach to identify pre and post-export interactors of an identified Plasmodium export element-negative exported protein named SBP1. To achieve this timely resolution, the authors chose to tag the endogenous SBP1 gene with the TurboID to allow shorter labeling periods and reduced nonspecific labeling compared to the original BirA variant as previously used to identify novel proteins of the Plasmodium mitochondrion (PMID: 35984838 - this reference must be added to the manuscript).

© 2023. Published by The Company of Biologists under the terms of the Creative Commons Attribution License (https://creativecommons.org/licenses/by/4.0/).

The authors first demonstrated that using immunofluorescence assays, the TurboID tag does not alter SBP1 trafficking and localization. Next, they showed no toxicity over four growth cycles in medium with Biotin. However, to reduce the background biotinylation, the authors took advantage of Plasmodium's ability to replicate in Biotin-free conditions asexually.

The authors performed 4 hours of biotin labeling on tightly synchronized early-ring stage parasites 4 or 20 hours post-infection to collect pre and post-exported proteins, respectively.

The authors identified 24 protein candidates as putative pre-export interactors including some known SBP1 interactors and a dozen uncharacterized proteins. It is unclear why the authors chose an IMC and putative mitochondrial protein for further characterization with the rationale that these two proteins might be putative translocon effectors to extract exported membrane proteins from the PPM. The revised manuscript must provide a more substantial justification or explanation for follow-up on these two proteins. In addition, most identified hits do not have TM domains, especially within the set of unknown function proteins. It is unclear whether the uncharacterized protein has predicted protein domains and homologs outside the Plasmodium genus. I would suggest the authors report a more detailed in silico analysis of their dataset to increase the impact and significance of the data. GAPM1 has already been shown to localize at the IMC in Plasmodium by Bullen et al. reference 56 in the current manuscript.

In contrast, GAPM1 proximity with EXP2 in the early-stage parasite is an important data of the manuscript that is poorly presented regarding the rationale of IMC proteins function in facilitating proteins export from the PPM. Because the study relies heavily on subcellular localization to validate putative pre-export interactors with SBP1, additional data at higher microscopic resolution using immuno-electron or expansion microscopy will significantly strengthen the study's findings. Overall the study is well robustly conducted with appropriate biological replicates and statistical analysis. In addition, the authors validated TurboID as a tool to study transient biological processes during the Plasmodium erythrocytic asexual replication. Finally, I recommend the authors revise the introduction section to increase visibility to nonexpert readers. In addition, I would suggest expanding the rationale on how IMC proteins might be critical to facilitate protein export from the PPM to the host cell. Post-invasion, how the IMC disassembly occurs is understudied and remains poorly understood. The authors briefly mentioned the hypothesis of a fusion event between the IMC and the PPM after the invasion. It would be interesting to discuss it in the context of the search for effectors of the PNEPS.

Comments for the author

The major revision concerns the characterization of GAPM1 And VAC subcellular localization in early-stage parasites. The IMC and PPM are 10 nm apart, so colocalization studies are challenging. The authors performed a colocalization study combined with SIM microscopy and demonstrated that GAPM1 is unlikely at the PVM; however, it would strengthen the study to determine whether GAPM1 is present at the PPM or PV. I recognize that immunoEM are challenging experiments and do not always yield precise results; however, the authors could try a more recently developed microscopy technique named expansion microscopy.

Minors comments:
Line 17: to increase clarity, I would recommend adding "to the host RBC" at the end of line 17.
" …how these exported membrane proteins are extracted from the PPM for export."
Line 40: not 2020 but 2021.
Line 50: The authors might want to describe PVM in more detail.
Line 72: Why do you mean by "there are no obvious candidates"
Line 75: Instead of proteomic approaches, I recommend writing the IP-based method, as mentioned later in the manuscript. Because proximal labeling is a proteomic approach and your study demonstrated that it is an appropriate tool to identify effectors of the translocon Line 90: I recommend the authors cite the recent work of Lamb et al. 2022 (PMID: 35984838), where they identified new proteins of the Plasmodium mitochondrion using TurboID.
Line 225: What data in figure 2C supports the localization of SBP1 in the ER?
Line 250: No immunostainings are supporting the localization of SBP1 at 20 hpi.

© 2023. Published by The Company of Biologists under the terms of the Creative Commons Attribution License (https://creativecommons.org/licenses/by/4.0/).

Line 331: Regarding the study published by Bullen et al. 2009 (reference 56 in the current manuscript), I would rewrite line 331 as " Our data confirmed that GAPM1...."
Line 332-333: Why do you reason that the "IMS most likely fuses to the PPM shortly after the invasion"? What data suggest you this rationale? What does it mean for the translocon?
Line 378-385: It is essential to remind the reader of the data in Supp figure 1.
I would add a line about Biotin-free media and discuss whether or not TurboID can only be used in biotin-free media.
Line 403: the 24 putative candidates are proximal to SBP1, and IP validation is required to tell whether or not they "interact" with SBP1. Therefore the authors must soften their language by saying proximal instead of interact.
Line 414: Do the authors have to assess SBP1 expression earlier than 48hr to claim that "achieving protein knockdown takes about 48hr? In addition, the KD might take only a few hours, but the SBP1 turnover might be the limiting factor.
Could the authors add some thoughts about it in their discussion?
Line 437: Regarding this sentence, the authors will correct line 331.
Line 443-444: Interesting speculations that I would like the authors to develop and justify. How could you test this hypothesis? What does it mean for other parasite stages where IMC is also disassembled after the invasion of their host cells? The authors might consider adding the review by Ferreira et al. 2020 (PMID: 33489940)

Figure 1:
I would recommend moving panel C to supplementary data and adding a schematic showing the integration of the repair plasmid to the targeted locus as done in Supp Figure 2 for the two other cell lines.
In Panel F, the phase contrast images are DIC pictures; if so, please indicate them. In addition, the quality is inconsistent, and the brightness could be increased.

Figure 2:
Panel A and B: I recommend the authors add the biotin concentration used Panel D: For the nonexpert in the field, I would recommend drawing a schematic representing the IFA data and labeling each subcellular compartment.

Figure 4:
Panel A: I recommend the authors add more details on the samples' biotin treatment and collection time.
Panel B: It would be informative to indicate the presence of homologs outside of the plasmodium genus.

Although the authors thoughtfully displayed separate channels in addition to the merged images. I would recommend the authors to rethink the choice of their representative colors for immunostaining and live-imaging regardless of the actual color of the fluorescent protein to make their figures accessible for color-blindness and to increase interpretation for everyone (the cyan color for TT staining made it challenging to interpret merges with green and avoid red especially in combination with green).
To help the authors see the below-suggested combination of colors:

- Magenta/green/blue
- Magenta/Yellow/Cyan
- Red/Cyan/Yellow

<u>Reviewer 3</u>

Advance summary and potential significance to field

Export of PEXEL-negative exported proteins (PNEPs), such as skeleton binding protein 1 (SBP1) and the major virulence protein, PfEMP1, underpins the pathology of P. falciparum malaria. The export process relies on a transmembrane (TM) domain that leads to membrane insertion at the ER and transfer into the secretory pathway. Extraction of membrane-embedded PNEPs from the parasite plasm membrane (PPM) for delivery to the Plasmodium translocon of exported proteins (PTEX)

© 2023. Published by The Company of Biologists under the terms of the Creative Commons Attribution License (https://creativecommons.org/licenses/by/4.0/).

occurs by an unknown process that is postulated to involve a putative Plasmodium translocon of exported membrane proteins (PTEM).

This manuscript from Vasant Muralidharan's lab describes the use of a promiscuous biotin ligase known as TurboID in an attempt to identify proteins that are physically close to SBP1 during its export path to the infected RBC host cell.

Comments for the author

The authors first examine the effect of incubation time on the TurboID labelling profile. Even in the absence of added biotin, weak bands are observed at ~48 kD 90 kD and 95 kD (see 2A left lane). The authors suggest that the 90 kD band is self-biotinylated SBP1. What is the evidence for that? Is the observed size consistent with the predicted molecular mass? It is of interest that the ~48 kD is at the expected size for unmodified SBP1. Is there some cleavage of the SBP1/TbID during preparation of samples for Western blotting?

The author use IFA (fog 2D) to demonstrate biotinylation in different regions of the infected RBC in rings (parasite periphery) and trophs (Maurer's clefts). They do not provide Western analyses of these different time points. Are the profiles different?

The authors undertook a proteomic analysis of streptavidin-precipitated proteins.
They provide Supp Table 2 with a list of all the proteins identified by mass spectrometry in the early and late samples. The authors identified 1,122 proteins, which represents about 50% of the total number of parasite proteins expressed in P. falciparum blood stage parasites. This is presumably a consequence of the high enzymatic activity of TurboID, which can lead to high levels of background labelling. The authors then faced the task of trying to distinguish relevant hits (i.e. proteins that are proximal to SBP1) from non-specifically biotinylated proteins.

The authors compared the proteins identified early in the export process (4 h p.i.) with those identified later (20 h pi.). The authors selected proteins with 10-fold higher abundance and 4 h compared with 20 h and propose that these represent interacting proteins within the PV compartment. The authors to assign 24 proteins to this category.

It is difficult to appreciate how the authors distinguish between proteins that are more highly expressed in ring stage than in troph stage from proteins that are more proximal to SBP1 in ring stage than in troph stage.

One observation from Table S2 is that SBP1 appears to be only ~100th in the list.
How does this correlate with the very intense band observed by Western?

Q8ILE3, a putative voltage-dependent anion-selective channel protein (VAC), was selected as a protein that was enriched in the early time point. Where there any peptides observed for VAC in the later sample? VAC appears to be a relatively low ranked biotinylated protein (~900th in abundance).

Fig 4C lists VAC as having no TM domain. Voltage-dependent anion channels (i.e. mitochondrial porins) are expected to form a beta barrel that spans the mitochondrial outer membrane. The transmembrane segments (amphipathic beta strands) would not be predicted by standard algorithms. In other organisms, VACs are synthesised as soluble proteins that spontaneously insert into the mitochondrial outer membrane. They do not need a mitochondrial signal to drive transport to the mitochondrion. In other organisms, VACs act as diffusion pore for small hydrophilic molecules, which is not the anticipated function for the putative PTEM.

The authors examined the location of VAC(apt) in ring stage transfectants. In the single IFA image that is presented (Fig 5A), the labelling profile is consistent with a mitochondrial location, as well as weaker labelling at the periphery.
Further images accompanied by image analysis would help provide confidence about the location. In the iamge presented, there is poor overlap with Exp2. While it remains formally possible that

© 2023. Published by The Company of Biologists under the terms of the Creative Commons Attribution License (https://creativecommons.org/licenses/by/4.0/).

VAC functions as part of the PTEM, taken alone the data provided do not provide strong evidence that VAC performs the proposed role.

The authors selected glideosome-associated protein with multiple membrane spans 1 (GAPM1) as another putative candidate. GAPM1 is a characterised protein of the inner membrane complex (IMC). The IMC is formed in P. falciparum segmented schizont stage. The authors suggest that the IMC is incorporated into the PM after invasion. Is there any EM evidence for this from other studies? IFA data suggest that GAPM1 is located at the parasite periphery in ring stage parasites – an interesting finding. However, again, the overlap with Exp2 is limited. Taken alone, these data do not provide strong evidence that VAC performs the role of the putative PTEM.

In summary, this is an interesting study that identifies many proteins that are potentially in the endomembrane (secretory pathway) and exomembrane (exported membrane structures). I feel as though the authors have missed an opportunity to undertake a more extensive analysis of the entire proteome identified in the work.

The authors have instead concentrated on two proteins that are enriched in the early stage biotinylated proteome. While these proteins are of interest, further data would be needed to support the authors claim that these proteins function as part of the putative PTEM. For example, evidence of accessibility to antibodies that recognise the HA tag, or to proteases, in saponin permeabilised cells might provide stronger evidence that the proteins really are located in the PPM, as requited to perform the role of the putative PTEM. One option may be to revise the claims that are made in the manuscript.

First revision

<u>Author response to reviewers' comments</u>

Reviewer 1 Advance Summary and Potential Significance to Field:

Summary
In this manuscript the authors pursue the so-called PTEM in P. falciparum by a novel and innovative time resolved, quantitative TurboID approach. The method clearly is time-resolved, but it is not clear whether meaningful interactions toward the elusive PTEM are identified as validations of two putative candidates, which are fairly long-shots at best, is inconclusive. To come up with a mechanistic model the authors need transition of proteins from the IMC to the PPM, which seems to be supported by the data. This by itself would be a mechanistic breakthrough that would be inconsistent with what is known about IMC disassembly, which is actually very little for the RBC stage. However, support for that is only partial at best. The VAC candidate suffers similar shortcomings.

Response: We thank the reviewer for the positive feedback. We agree that the data do not conclusively prove that the identified candidates are part of the elusive PTEM. However, these are the only candidates that were identified and the controls suggest that this new tool is working as defined to identify these transient interactors. Therefore, we have edited the manuscript to tone down our conclusions. The resubmitted manuscript includes new movies showing the IMC in newly invaded merozoites as well as expansion microscopy showing that the IMC markers are in the parasite periphery within newly invaded rings, suggesting that it fuses to the parasite plasma membrane. We have also included new data showing that both GAPM1 and VAC co-localize with another exported membrane protein, MAHRP1 in early ring stage parasites (Fig. 5 C,D). These data are consistent with the model that both candidates facilitate export of membrane proteins during ring stages. Other specific comments of the reviewer are addressed below.

Reviewer 1 Comments for the Author:
Specific points
1. Line 332 (and 438/9, 444). “we reasoned that the IMC most likely fuses to the PPM shortly after

© 2023. Published by The Company of Biologists under the terms of the Creative Commons Attribution License (https://creativecommons.org/licenses/by/4.0/).

invasion". This is a pretty bold assumption without any solid basis. At least in the Plasmodium sporozoite (Coppens lab and Meis et al in the 1980s: IMC detached from the plasma membrane accumulates in the cytoplasm) and Toxoplasma (various labs: very localized breakdown in apical to basal direction) some insights have been generated in how organized and coordinated these processes are, at least by electron microscopy. Admittedly, no mechanistic details are known, but assuming PPM fusion would dramatically expand the parasite within minutes post invasion, which does not seem to be consistent with observations made in merozoites (Gilberger lab: 15 min after completing invasion only small IMC membrane pieces remain (Riglar et al., 2013). Indeed, the presented data seem to hint at (partial or selective) fusion of IMC and PPM to some extend (or translocation of some IMC proteins?), but even that would need to be further validated.

Response: We have edited our conclusions. The previous data does not show what happens to the IMC, only that it seems to disappear. These prior data also do not show what happens to the IMC lipids or IMC proteins (Ref. 54).

While we agree with the reviewer that previous observations have shown that the IMC is seen as small pieces, the parasite does dramatically increase in size post-invasion. This has been observed as amoeboid movements of the parasite post-invasion to form ring stages, which are bigger than merozoites. However, as the reviewer correctly points out, our data is not enough to overturn the existing model. Our data is consistent with two possible models, one where the IMC fuses to the plasma membrane and another where GAPM1 specifically relocalizes from the IMC to the PPM. The newly included microscopy as well as live imaging data (Figs. 5 and 6) are consistent with both models. The conclusions have been edited to reflect this (lines 387-391 and 488-490).

2. The presented data do not permit to associate any of the identified candidates directly with the elusive PTEM. The presented hypothetical scenarios are tantalizing, but require a lot of assumptions and handwaving, and certainly the suggested DD or other faster kinetic protein knock-down would be needed to support these scenarios

Response: In the nearly two decades since the PTEM was first proposed, no candidates have been identified. Therefore, we developed a novel tool to identify candidates for this proposed translocon. New expansion microscopy data (Fig. 6) and co-localization data suggest that the proposed candidates co-localize with the exported membrane protein, MAHRP2 (Fig. 5). Together, these data allow us to suggest that the identified candidates may play a role in export of these proteins. Future work will help define the exact mechanistic function of these candidates in export of membrane proteins.Therefore, we have edited the resubmitted manuscript to be more careful in our conclusions and refrain from calling the identified candidates as the putative PTEM.

Minor points
1. Line 239: Fig. 3C should be 3B
   Response: Corrected.
2. Lines 256, 263. The "square" referred to is a rectangle. "box" might be an alternative term that is more astute.
   Response: Changed.

Reviewer 2 Advance Summary and Potential Significance to Field:
Review of "
Time resolved proximity biotinylation implicates a porin domain protein in export of malaria parasite effectors"

My recommendation is acceptance of the paper with one major and minor revisions.

Response: We thank the reviewer for their careful evaluation of our manuscript. The specific comments of the reviewer are addressed below.

Despite recent advances in vaccine development, malaria remains a worldwide public health issue with the rapid emergence of resistance to the frontline artemisinin combination therapy.

© 2023. Published by The Company of Biologists under the terms of the Creative Commons Attribution License (https://creativecommons.org/licenses/by/4.0/).

In addition, clinical symptoms of malaria begin with the invasion and proliferation of Plasmodium parasites in red blood cells. Therefore a better understanding at the molecular level of the parasitic biological processes required for parasite invasion, growth, and egress from erythrocytes will likely facilitate the development of novel antimalarial drugs.

One attractive target is to disrupt the parasite protein export pathway. To proliferate, Plasmodium falciparum, the deadliest causing agent of malaria, must remodel the erythrocytic plasma membrane through the export of hundreds of proteins across the parasitophorous vacuole membrane using the Plasmodium translocon of exported proteins or PTEX. However, how membrane proteins are extracted from the parasite plasma membrane and delivered to the PTEX complex remains poorly understood at the molecular level.

In this study, the authors took a proximal labeling-based approach to identify pre and post-export interactors of an identified Plasmodium export element- negative exported protein named SBP1. To achieve this timely resolution, the authors chose to tag the endogenous SBP1 gene with the TurboID to allow shorter labeling periods and reduced nonspecific labeling compared to the original BirA variant as previously used to identify novel proteins of the Plasmodium mitochondrion (PMID: 35984838 - this reference must be added to the manuscript).

Response: We agree with the reviewer and have included the BioRXiv preprint version of the reference. The reference has been updated now.

The authors first demonstrated that using immunofluorescence assays, the TurboID tag does not alter SBP1 trafficking and localization. Next, they showed no toxicity over four growth cycles in medium with Biotin. However, to reduce the background biotinylation, the authors took advantage of Plasmodium's ability to replicate in Biotin-free conditions asexually.
The authors performed 4 hours of biotin labeling on tightly synchronized early- ring stage parasites 4 or 20 hours post-infection to collect pre and post- exported proteins, respectively. The authors identified 24 protein candidates as putative pre-export interactors, including some known SBP1 interactors and a dozen uncharacterized proteins. It is unclear why the authors chose an IMC and putative mitochondrial protein for further characterization with the rationale that these two proteins might be putative translocon effectors to extract exported membrane proteins from the PPM. The revised manuscript must provide a more substantial justification or explanation for follow-up on these two proteins.

Response: We chose these two proteins based on the criteria that to extract membrane proteins, the putative candidate proteins must localize to the membrane. These were the only two candidates in the pre-export fraction that fulfilled these criteria. We have included this rationale in the resubmitted manuscript.

In addition, most identified hits do not have TM domains, especially within the set of unknown function proteins. It is unclear whether the uncharacterized protein has predicted protein domains and homologs outside the Plasmodium genus. I would suggest the authors report a more detailed in silico analysis of their dataset to increase the impact and significance of the data. GAPM1 has already been shown to localize at the IMC in Plasmodium by Bullen et al. reference 56 in the current manuscript.
In contrast, GAPM1 proximity with EXP2 in the early-stage parasite is an important data of the manuscript that is poorly presented regarding the rationale of IMC proteins function in facilitating proteins export from the PPM. Because the study relies heavily on subcellular localization to validate putative pre- export interactors with SBP1, additional data at higher microscopic resolution using immuno-electron or expansion microscopy will significantly strengthen the study's findings. Overall the study is well robustly conducted with appropriate biological replicates and statistical analysis. In addition, the authors validated TurboID as a tool to study transient biological processes during the Plasmodium erythrocytic asexual replication. Finally, I recommend the authors revise the introduction section to increase visibility to nonexpert readers. In addition, I would suggest expanding the rationale on how IMC proteins might be critical to facilitate protein export from the PPM to the host cell. Post-invasion, how the IMC disassembly occurs is understudied and remains poorly understood. The authors briefly mentioned the hypothesis of a fusion event between the IMC and the PPM after the invasion. It would be interesting to discuss it in the context of the search for effectors of

© 2023. Published by The Company of Biologists under the terms of the Creative Commons Attribution License (https://creativecommons.org/licenses/by/4.0/).

the PNEPS.

Response: We thank the reviewer for recognizing the robustness of this study. As suggested, we have utilized ultrastructural expansion microscopy to gain more insights into the localization of the candidates (Fig. 6) as well as observed GAPM1 post-invasion via live fluorescence microscopy (Fig. 6). These studies helped us refine our model as suggested by this and other reviewers.

Reviewer 2 Comments for the Author:
The major revision concerns the characterization of GAPM1 And VAC subcellular localization in early-stage parasites. The IMC and PPM are 10 nm apart, so colocalization studies are challenging. The authors performed a colocalization study combined with SIM microscopy and demonstrated that GAPM1 is unlikely at the PVM; however, it would strengthen the study to determine whether GAPM1 is present at the PPM or PV. I recognize that immunoEM are challenging experiments and do not always yield precise results; however, the authors could try a more recently developed microscopy technique named expansion microscopy.

Response: As suggested by the reviewer, we used U-ExM to localize VAC and GAPM1. The data are consistent with the model that they localize closely with the PV markers (RAP1) at the parasite periphery (Fig. 6). However, as predicted by the reviewer, the resolution was insufficient to completely differentiate between the PPM and PVM. Our data suggest that GAPM1 re-localizes to the parasite periphery or the IMC fuses with the PPM. The data are unable to differentiate between the two models and another set of experiments would be required to do so, which would be outside the scope of this manuscript.

Minors comments:

Line 17: to increase clarity, I would recommend adding "to the host RBC" at the end of line 17.
" ...how these exported membrane proteins are extracted from the
PPM for export."
Response: Added.

Line 40: not 2020 but 2021.
Response: Changed.

Line 50: The authors might want to describe PVM in more detail.
Response: Added.

Line 72: Why do you mean by "there are no obvious candidates"

Response: Changed to '..no candidates have been identified using bioinformatic approaches..'.

Line 75: Instead of proteomic approaches, I recommend writing the IP-based method, as mentioned later in the manuscript. Because proximal labeling is a proteomic approach and your study demonstrated that it is an appropriate tool to identify effectors of the translocon

Response: Changed.

Line 90: I recommend the authors cite the recent work of Lamb et al. 2022 (PMID: 35984838), where they identified new proteins of the Plasmodium mitochondrion using TurboID.

Response: We have cited this paper later in the manuscript.

Line 225: What data in figure 2C supports the localization of SBP1 in the ER?

Response: We have clarified it, we did not mean SBP1 is in the ER but rather that their synthesis occurs in the ER as all proteins in the secretory pathway are synthesized into the rough ER.

Line 250: No immunostainings are supporting the localization of SBP1 at 20 hpi.

Response: We cite Fig. 2D now, showing SBP1 is at the MC at 20hpi, as well as cite McMillan et al

© 2023. Published by The Company of Biologists under the terms of the Creative Commons Attribution License (https://creativecommons.org/licenses/by/4.0/).

(ref. 49) who also observed SBP1 at the MC at 20hpi.

Line 331: Regarding the study published by Bullen et al. 2009 (reference 56 in the current manuscript), I would rewrite line 331 as " Our data confirmed that GAPM1…."

Response: Changed.

Line 332-333: Why do you reason that the "IMS most likely fuses to the PPM shortly after the invasion"? What data suggest you this rationale? What does it mean for the translocon?

Response: As before, we have changed this. Our data suggest that GAPM1 relocalizes to the parasite periphery after invasion and previous work (Riglar et al 2013) suggests that the IMC is broken down quickly after invasion. Hence, our observations are inconsistent with that finding, though our data cannot rule out that GAPM1 alone relocalizes to the PPM while the rest of the IMC is degraded as previously suggested. We have edited this in the results as well as in the discussion to clarify our data and our conclusions.

Line 378-385: It is essential to remind the reader of the data in Supp figure 1.
I would add a line about Biotin-free media and discuss whether or not TurboID can only be used in biotin-free media.

Response: Added.

Line 403: the 24 putative candidates are proximal to SBP1, and IP validation is required to tell whether or not they "interact" with SBP1. Therefore the authors must soften their language by saying proximal instead of interact.

Response: Changed.

Line 414: Do the authors have to assess SBP1 expression earlier than 48hr to claim that "achieving protein knockdown takes about 48hr? In addition, the KD might take only a few hours, but the SBP1 turnover might be the limiting factor. Could the authors add some thoughts about it in their discussion?

Response: SBP1 is made early in the lifecycle and exported in a bolus as our data (Figs.1-3) shows, which is consistent with previously published work. Once it reaches the RBC (~6-8 hpi), it then stays at the Maurer's clefts for the rest of the asexual life cycle. In the new lifecycle, it is then remade and exported. Since our knockdown takes >24h and parasites die before they reach the next lifecycle, we cannot assess the effect of knockdown on export because the SBP1 is already at the Maurer's clefts and no more SBP1 is made. We have added further clarification in the discussion.

Line 437: Regarding this sentence, the authors will correct line 331.
Response: Done.

Line 443-444: Interesting speculations that I would like the authors to develop and justify. How could you test this hypothesis? What does it mean for other parasite stages where IMC is also disassembled after the invasion of their host cells? The authors might consider adding the review by Ferreira et al. 2020 (PMID: 33489940)

Response: We thank the reviewer for this suggestion and have added the reference. The discussion has been expanded to answer these questions (lines 479-490)

Figure 1:
I would recommend moving panel C to supplementary data and adding a schematic showing the integration of the repair plasmid to the targeted locus as done in Supp Figure 2 for the two other cell lines.

In Panel F, the phase contrast images are DIC pictures; if so, please indicate them. In addition,

© 2023. Published by The Company of Biologists under the terms of the Creative Commons Attribution License (https://creativecommons.org/licenses/by/4.0/).

the quality is inconsistent, and the brightness could be Increased.

Response: We apologize for the loss of resolution in the PDF. Higher resolution images were used and will be provided for publication. The repair plasmids and integration schematic have been added to Fig. S2.

Figure 2:
Panel A and B: I recommend the authors add the biotin concentration used Panel D: For the nonexpert in the field, I would recommend drawing a schematic representing the IFA data and labeling each subcellular compartment.

Response: The biotin concentrations have been added to the figure legend.The schematic has been included in figure 3.

Figure 4:
Panel A: I recommend the authors add more details on the samples' biotin treatment and collection time.
Panel B: It would be informative to indicate the presence of homologs outside of the plasmodium genus.

Response: Details have been included in the resubmission.

Although the authors thoughtfully displayed separate channels in addition to the merged images. I would recommend the authors to rethink the choice of their representative colors for immunostaining and live-imaging regardless of the actual color of the fluorescent protein to make their figures accessible for color-blindness and to increase interpretation for everyone (the cyan color for TT staining made it challenging to interpret merges with green and avoid red, especially in combination with green).
To help the authors see the below-suggested combination of colors:

- Magenta/green/blue
- Magenta/Yellow/Cyan
- Red/Cyan/Yellow

Response: We thank the reviewer for bringing this issue to our attention. We have made the appropriate changes and all IFAs have been re-colored to make it more accessible to everyone.

Reviewer 3 Advance Summary and Potential Significance to Field:
Export of PEXEL-negative exported proteins (PNEPs), such as skeleton binding protein 1 (SBP1) and the major virulence protein, PfEMP1, underpins the pathology of P. falciparum malaria. The export process relies on a transmembrane (TM) domain that leads to membrane insertion at the ER and transfer into the secretory pathway. Extraction of membrane-embedded PNEPs from the parasite plasm membrane (PPM) for delivery to the Plasmodium translocon of exported proteins (PTEX) occurs by an unknown process that is postulated to involve a putative Plasmodium translocon of exported membrane proteins (PTEM).

This manuscript from Vasant Muralidharan's lab describes the use of a promiscuous biotin ligase known as TurboID in an attempt to identify proteins that are physically close to SBP1 ^{Tb} during its export path to the infected RBC host cell.
Response: We appreciate the reviewers' positive evaluation of our manuscript and have addressed their specific comments below.

Reviewer 3 Comments for the Author:
The authors first examine the effect of incubation time on the TurboID labelling profile. Even in the absence of added biotin, weak bands are observed at ~48 kD, 90 kD and 95 kD (see 2A left lane). The authors suggest that the 90 kD band is self-biotinylated SBP1. What is the evidence for that? Is the observed size consistent with the predicted molecular mass? It is of interest that the ~48 kD is at the expected size for unmodified SBP1. Is there some cleavage of the SBP1/TbID during preparation of samples for Western blotting?

Response: The V5 peptide tag is also present in SBP1^{TbID} and is shown on the western blot. It co-

© 2023. Published by The Company of Biologists under the terms of the Creative Commons Attribution License (https://creativecommons.org/licenses/by/4.0/).

localizes with the streptavidin band at (90 kD) and helps us determine that SBP1^{TbID} is likely self-biotinylating. The observed size is consistent with the molecular mass of the fusion protein (SBP1 ~48kDa+ TurboID ~36kDa+ linker and V5 tag ~2kDa= 86kDa). We don't see evidence of the 48kDa band in the V5 blot, so it is likely another protein.

The author use IFA (fog 2D) to demonstrate biotinylation in different regions of the infected RBC in rings (parasite periphery) and trophs (Maurer's clefts). They do not provide Western analyses of these different time points. Are the profiles Different?

Response: The proteomic data provides a better resolution of the time points than a Western blots. A single Western band usually contains several proteins since the separation is only done in one dimension. Hence, we did not use Western analysis to identify specific bands at these time points. It is very difficult to identify profile differences on a Western blot by the naked eye and this would also introduce operator bias into the system, which we wanted to avoid. Since we are interested in transient interactions between the secreted membrane proteins and their transporters, we reasoned that this proximal labeling approach would be ideal.

The authors undertook a proteomic analysis of streptavidin-precipitated proteins. They provide Supp Table 2 with a list of all the proteins identified by mass spectrometry in the early and late samples. The authors identified 1,122 proteins, which represents about 50% of the total number of parasite proteins expressed in P. falciparum blood stage parasites. This is presumably a consequence of the high enzymatic activity of TurboID, which can lead to high levels of background labelling. The authors then faced the task of trying to distinguish relevant hits (i.e. proteins that are proximal to SBP1) from non- specifically biotinylated proteins.

Response: We agree that the high number of proteins identified probably reflects the high enzymatic activity of TurboID. Hence, we undertook this quantitative approach to identify proximal proteins that are identified reproducibly with high statistical confidence (Fig. 4).

The authors compared the proteins identified early in the export process (4 h p.i.) with those identified later (20 h pi.). The authors selected proteins with 10-fold higher abundance and 4 h compared with 20 h and propose that these represent interacting proteins within the PV compartment. The authors to assign 24 proteins to this category.

It is difficult to appreciate how the authors distinguish between proteins that are more highly expressed in ring stage than in troph stage from proteins that are more proximal to SBP1 in ring stage than in troph stage.

Response: We appreciate the reviewer bringing this up. We only have RNA expression data for the different stages, which does not always correspond to protein levels. For example, EXP2 (RNA expressed in troph stages) and HSP101, components of the export translocon on the PVM, are exceptionally abundant proteins in the ring stages. However, these do not show up as reproducibly as proteins proximal to SBP1 even though they are in the secretory pathway and are required for protein export. Similarly, digestive vacuole proteases are very abundant proteins in the secretory pathway during the trophozoite stage but these do not show up reproducibly on our list. This study relied on the time-resolution aspect along with quantitative proteomics to identify SBP1 proximal proteins during its export. If we re-parse the data to include or exclude proteins putatively expressed in one stage or another using RNA expression profiles, we reintroduce operator bias into the experiment.

One observation from Table S2 is that SBP1 appears to be only ~100th in the list. How does this correlate with the very intense band observed by Western?

Response: Where a protein is on the list is not relevant because this is a quantitative proteomic approach that relies on several biological replicates. The other factor to consider here is that unlike an IP, the proximity biotinylation approach relies on self-biotinylation to pull down the bait protein. The level of self-biotinylation would be variable and cannot be controlled. It is not clear how one would correlate Western band intensity with 'rank'. A single Western band may (and usually does) contain several dozen proteins since the separation is only in one dimension. While identifying a protein by mass spectrometry relies on separation over 2-4 dimensions, one during

© 2023. Published by The Company of Biologists under the terms of the Creative Commons Attribution License (https://creativecommons.org/licenses/by/4.0/).

the liquid chromatography and another during the two time-of-flight detectors and yet another by ion fragmentation. This enables mass spectrometry to be several orders of magnitude more sensitive than a Western. We further added a time-dimension to our approach and developed this approach to obtain an unbiased look at the proteins proximal to SBP1. Using simply band 'intensity' on a Western blot would lead to a more biased approach and will introduce operator bias into the system.

Q8ILE3, a putative voltage-dependent anion-selective channel protein (VAC), was selected as a protein that was enriched in the early time point. Where there any peptides observed for VAC in the later sample? VAC appears to be a relatively low ranked biotinylated protein (~900th in abundance).

Response: Using 'rank' to determine the importance of a protein is misleading because the 'rank' does not take into account the biological reproducibility (as determined by the p-value on the volcano plot) or the identification of the protein at the specific time point (shown as fold change on the volcano plot). Using this proximal labeling approach and doing several biological replicates helps us build a more accurate and unbiased list. The fact that we reproducibly detected a low abundance protein and it was only enriched at the early time point demonstrates the utility of this approach. VAC was detected in all biological replicates at the 4h time point and not reproducibly detected at 20h.

Fig 4C lists VAC as having no TM domain. Voltage-dependent anion channels (i.e. mitochondrial porins) are expected to form a beta barrel that spans the mitochondrial outer membrane. The transmembrane segments (amphipathic beta strands) would not be predicted by standard algorithms. In other organisms, VACs are synthesised as soluble proteins that spontaneously insert into the mitochondrial outer membrane. They do not need a mitochondrial signal to drive transport to the mitochondrion. In other organisms, VACs act as diffusion pore for small hydrophilic molecules, which is not the anticipated function for the putative PTEM.

Response: We agree with the reviewer that VAC likely forms a beta barrel spanning the membrane, hence we chose to follow up on VAC. While it is true that a mitochondrial targeting sequence may not be required, *P. falciparum* does have another ortholog of VAC that DOES have a MTS. Furthermore, Dr. Vaidya's group (Ref. 68, Lamb et al) independently used the same proximity biotinylation approach to identify mitochondrial proteins. They detected the VAC ortholog with the targeting sequence in the mitochondria. Their approach also detected the VAC we identified but only in the non-mitochondrial pellet fraction, which they determined to consist of other membranes such as the PPM (lines 317-318 and 467-471).

The authors examined the location of VAC(apt) in ring stage transfectants. In the single IFA image that is presented (Fig 5A), the labelling profile is consistent with a mitochondrial location, as well as weaker labelling at the periphery.

Further images accompanied by image analysis would help provide confidence about the location. In the iamge presented, there is poor overlap with Exp2. While it remains formally possible that VAC functions as part of the PTEM, taken alone, the data provided do not provide strong evidence that VAC performs the proposed Role.

Response: Maybe the reviewer missed this but along with the image, we did provide quantification (Pearson's correlation coefficient) from several images showing VAC colocalizes with EXP2, a PV marker (Fig. 5B). This has been bolstered with new data showing co-localization via ultrastructural expansion microscopy images (Fig. 6), as well as showing co-localization with another exported MC protein, MAHRP1 (Fig. 5C,D). Super-resolution microscopy showing the same localization is now shown in Supplementary Figure 4.

We agree that this is not conclusive evidence but it is consistent with our model. We have modified our conclusions both in the results and discussion section to reflect this.

The authors selected glideosome-associated protein with multiple membrane spans 1 (GAPM1) as another putative candidate. GAPM1 is a characterised protein of the inner membrane complex (IMC). The IMC is formed in P. falciparum segmented schizont stage. The authors suggest that the IMC is incorporated into the PM after invasion. Is there any EM evidence for this from other studies?

© 2023. Published by The Company of Biologists under the terms of the Creative Commons Attribution License (https://creativecommons.org/licenses/by/4.0/).

Response: EM data suggests that the IMC disappears quickly after invasion, though it is not known what happens to the IMC membrane or the IMC proteins (Ref. 54 and lines 488-492). Surprisingly, prior work did not observe the fate of IMC proteins post-invasion. Our study is the first to show what happens to an IMC protein post-invasion. Our data shows that GAPM1 is present at the parasite periphery in early rings. These data are consistent with two models. In one model, the IMC fuses with the PPM (perhaps during the amoeboid stage?) to rapidly increase the size of the ring stage parasites. Another model would be that GAPM1 relocalizes to the PPM while the rest of the IMC is degraded. Our data are consistent with both models and we have edited our conclusions stating the same (lines 3896-391 and482-492).

IFA data suggest that GAPM1 is located at the parasite periphery in ring stage parasites – an interesting finding. However, again, the overlap with Exp2 is limited. Taken alone, these data do not provide strong evidence that VAC performs the role of the putative PTEM.

Response: We agree, hence we also show that both GAPM1 and VAC also co-localize strongly with another exported membrane protein, MAHRP1 (Fig. 5 C,D). We have also included new ultrastructural expansion microscopy data showing that both proteins are in close juxtaposition with a PV lumen marker, RAP1 (Fig. 6). These data are consistent with our model and the conclusions have been revised accordingly.

In summary, this is an interesting study that identifies many proteins that are potentially in the endomembrane (secretory pathway) and exomembrane (exported membrane structures). I feel as though the authors have missed an opportunity to undertake a more extensive analysis of the entire proteome identified in the Work.

Response: We thank the reviewer for this suggestion. While we agree that there are several proteins here that we will follow up on. Given the lack of clear localization signals on several of the uncharacterized and apicomplexan specific proteins, we would need to tag all these proteins to extensively study their functions and localizations. However that would be beyond the scope of one study.
The authors have instead concentrated on two proteins that are enriched in the early stage biotinylated proteome. While these proteins are of interest, further data would be needed to support the authors claim that these proteins function as part of the putative PTEM. For example, evidence of accessibility to antibodies that recognise the HA tag, or to proteases, in saponin permeabilised cells might provide stronger evidence that the proteins really are located in the PPM, as requited to perform the role of the putative PTEM. One option may be to revise the claims that are made in the manuscript.

Response: We agree with the reviewer that more work will be needed to establish the identified proteins as a bonafide PTEM. As the reviewer is well aware, the PTEX was identified first as a complex at the right place at the right time. It took almost a decade's worth of work to establish the PTEX as a bonafide translocon. Hence, we have revised the conclusions of our study accordingly.

Second decision letter

MS ID#: JOCES/2022/260506

MS TITLE: TIME-RESOLVED PROXIMITY BIOTINYLATION DURING PROTEIN EXPORT IMPLICATES A PORIN DOMAIN PROTEIN IN EXPORT OF TANSMEMBRANE MALARIA PARASITE EFFECTORS
AUTHORS: David Anaguano, Watcharatip Dedkhad, Carrie F Brooks, David W Cobb, and Vasant Muralidharan
ARTICLE TYPE: Research Article

We have now reached a decision on the above manuscript.

© 2023. Published by The Company of Biologists under the terms of the Creative Commons Attribution License (https://creativecommons.org/licenses/by/4.0/).

To see the reviewers' reports and a copy of this decision letter, please go to: https://submit-jcs.biologists.org and click on the 'Manuscripts with Decisions' queue in the Author Area. (Corresponding author only has access to reviews.)

As you will see, the reviewers gave favourable reports and we would like to accept your paper. Before doing so, however, I ask you address the minor point from reviewer 2 related to DNA staining to be sure about the parasite stages. Once you address this point, we would be pleased to publish your work.

Reviewer 1

Advance summary and potential significance to field

The advances are the successful establishment of spatial/time-resolved TurboID, the mapping of putatively PTEM relevant candidates, and hints at a totally new mechanism on the fate of the IMC post-invasion, which has the potential to opening up a new field and establish new concepts.

Comments for the author

The exciting new microscopy data now included in the manuscript, together with the rebuttal wherein the ameboid behavior shortly after invasion is tentatively linked to IMC fusion with the PM has me hooked. I think there is something real going on here, and this work might be the start of a new research direction, which might not be PTEM, although that route is still open. The arguments for which candidates to follow up are still debatable, but they are sufficiently justified. In combination with the successful establishment of spatial/time-resolved TurboID, this is now a more mature manuscript.

Reviewer 2

Advance summary and potential significance to field

The revised manuscript responds to my comments and I commend the authors for the additional expansion and live microscopy data. The current manuscript is suitable for publication once the figure 6 comment is addressed

Comments for the author

Minor Comment: In Figure 6, Panel A, the authors have displayed early ring-stage parasites at 4 hpi. However, the absence of DNA dye staining raises some uncertainty about whether these parasites are indeed in the early ring stage.
Additionally, based on the NHS-ester staining, it is reasonable to argue that the first image represents a 2N trophozoite, as evidenced by the strong staining of the two MTOCs. The third image appears somewhat atypical even for an ameboid-shaped ring, which is typically observed at 4 hpi. Lastly, the fourth parasite exhibits a distinct large food vacuole with multiple MTOCs, further suggesting that we may be looking at young trophozoites rather than young rings. It would be beneficial for the authors to include DNA staining to eliminate any ambiguity in the characterization of these parasites.

Reviewer 3

Advance summary and potential significance to field

The authors have addressed my queries.

Comments for the author

The authors have addressed my queries.

© 2023. Published by The Company of Biologists under the terms of the Creative Commons Attribution License (https://creativecommons.org/licenses/by/4.0/).

Second revision

Author response to reviewers' comments

Response to Reviewers
Reviewer 1 Advance Summary and Potential Significance to Field:
The advances are the successful establishment of spatial/time-resolved TurboID, the mapping of putatively PTEM relevant candidates, and hints at a totally new mechanism on the fate of the IMC post-invasion, which has the potential to opening up a new field and establish new concepts.

Reviewer 1 Comments for the Author:
The exciting new microscopy data now included in the manuscript, together with the rebuttal wherein the ameboid behavior shortly after invasion is tentatively linked to IMC fusion with the PM has me hooked. I think there is something real going on here, and this work might be the start of a new research direction, which might not be PTEM, although that route is still open. The arguments for which candidates to follow up are still debatable, but they are sufficiently justified. In combination with the successful establishment of spatial/time-resolved TurboID, this is now a more mature manuscript.

Response: We thank the reviewer for recognizing the exciting new avenues our work presents as well as outlining some valid concerns. Ongoing work will help answer some of the questions and help resolve these debates.

Reviewer 2 Advance Summary and Potential Significance to Field:
The revised manuscript responds to my comments and I commend the authors for the additional expansion and live microscopy data. The current manuscript is suitable for publication once the figure 6 comment is addressed

Response: We thank the reviewer for their positive comments and their support of our work.

Reviewer 2 Comments for the Author:

Minor Comment: In Figure 6, Panel A, the authors have displayed early ring-stage parasites at 4 hpi. However, the absence of DNA dye staining raises some uncertainty about whether these parasites are indeed in the early ring stage. Additionally, based on the NHS-ester staining, it is reasonable to argue that the first image represents a 2N trophozoite, as evidenced by the strong staining of the two MTOCs. The third image appears somewhat atypical even for an ameboid-shaped ring, which is typically observed at 4 hpi. Lastly, the fourth parasite exhibits a distinct large food vacuole with multiple MTOCs, further suggesting that we may be looking at young trophozoites rather than young rings. It would be beneficial for the authors to include DNA staining to eliminate any ambiguity in the characterization of these parasites.

Response: We are not sure how the reviewer is observing MTOCs in the parasites show in in Figure 6. It is extremely unlikely that these are anything other than rings since these parasites were synchronized using Percoll purified schizonts that were allowed to invade for 4 hours and all remaining schizonts were lysed using sorbitol lysis. These rings were then immediately fixed for expansion microscopy. Thus, it would be exceptionally unlikely that there are any trophozoites that are present (which require ~12 more hours to develop). If there were any 2N trophozoites present, they would have been lysed by sorbitol.

We have included DNA staining in Figure 6A to allay these fears as well.

Reviewer 3 Advance Summary and Potential Significance to Field:
The authors have addressed my queries.

© 2023. Published by The Company of Biologists under the terms of the Creative Commons Attribution License (https://creativecommons.org/licenses/by/4.0/).

Reviewer 3 Comments for the Author:
The authors have addressed my queries.

Response: Thank you.

Third decision letter

MS ID#: JOCES/2022/260506
Second decision letter

MS ID#: JOCES/2022/260506

MS TITLE: TIME-RESOLVED PROXIMITY BIOTINYLATION DURING PROTEIN EXPORT IMPLICATES A PORIN DOMAIN PROTEIN IN EXPORT OF TRANSMEMBRANE MALARIA PARASITE EFFECTORS

AUTHORS: David Anaguano, Watcharatip Dedkhad, Carrie F Brooks, David W Cobb, and Vasant Muralidharan
ARTICLE TYPE: Research Article

I am happy to tell you that your manuscript has been accepted for publication in Journal of Cell Science, pending standard ethics checks.

© 2023. Published by The Company of Biologists under the terms of the Creative Commons Attribution License (https://creativecommons.org/licenses/by/4.0/).
